# Stimuli-Responsive Hydrogels from Liquid–Liquid Phase Separations of FUS-Derived Peptides

**DOI:** 10.1021/acsami.5c15249

**Published:** 2025-09-24

**Authors:** Elisabetta Rosa, Mariantonietta Pizzella, Luca Cimmino, Valeria Castelletto, Ian W. Hamley, Luigi Vitagliano, Alfonso De Simone, Antonella Accardo

**Affiliations:** † Department of Pharmacy, Research Centre on Bioactive Peptides (CIRPeB), 9307 University of Naples “Federico II”, Via De Amicis 95, Naples 80145, Italy; ‡ IRCCS SYNLAB SDN, Via Ferraris 144, Naples 80146, Italy; § School of Chemistry, Pharmacy and Food Biosciences, 6816 University of Reading, Berkshire RG6 6AD, U.K.; ∥ Institute of Biostructures and Bioimaging, CNR, Via Castellino 111, Napless 80131, Italy

**Keywords:** stimuli-responsive hydrogels, low-complexity aromatic-rich kinked segments (LARKS), protein liquid–liquid phase separations (LLPS), biomaterials, rheological characterization

## Abstract

Defining soft biomaterials, including stimuli-responsive hydrogels, is essential for advancing applications such as targeted drug delivery, biosensing, and tissue engineering due to their ability to respond to environmental triggers dynamically. In this study, we characterized phase-separating peptides and elucidated the principles governing their self-assembly into hydrogels. Low-complexity aromatic-rich kinked segments (LARKS) were employed as building blocks to generate stimuli-responsive materials. By analyzing the properties of various multi-LARKS peptides, we developed a model informing the rational design of point mutations to modulate the mechanical properties and temperature stability of LARKS-based hydrogels, resulting in stimuli-responsive matrices. Our findings were further supported by demonstrating that these hydrogels effectively act as reservoir matrices capable of releasing drugs efficiently at 40 °C, highlighting their potential for biotechnological and medical applications.

## Graphical abstract

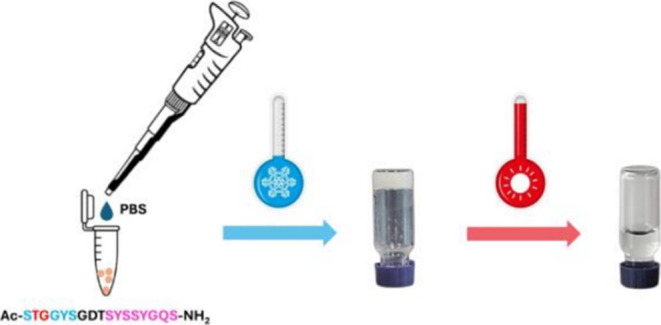

## Introduction

Protein liquid–liquid phase separations (LLPS) are emerging as ubiquitous phenomena in eukaryotic cells. These condensates are promoted by complex, multivalent biomolecular interactions driving the formation of membrane-less organelles and organizing cellular functions such as stress granule assembly, immune signaling, transcription, autophagy, and compartmentalization.^1^ LLPS have also been found in association with pathological conditions leading to protein self-assembly into amyloids in the context of neurodegenerative disorders, or in conjunction with inflammatory diseases and cancer.^2,3^ In the case of frontotemporal lobar degeneration (FTLD),^4^ it was demonstrated that the arginine hypomethylation in the Fused in Sarcoma (FUS) protein engages in aberrant condensation, causing the disruption of ribonucleoprotein (RNP) granule function and the inhibition of new protein synthesis in neuron terminals. Studies of the biological self-assembly through which these complex structures are formed in vivo have led scientists to the intuition that the interaction motifs of phase-separating proteins could be exploited to promote de novo design of new artificial materials. Harnessing LLPS-mediated pathways provides unique opportunities to design peptide-based scaffolds with tunable morphologies and advantageous properties for advanced biomedical applications.^5,6^ Similar to successes obtained with amyloid peptides,^7–9^ the identification of minimal sequences that are capable of mimicking the behavior of their parental proteins may provide valuable structural information on complex systems. For example, recent studies identified glycine-, serine- and tyrosine-rich Low Complexity Domains (LCDs)^10^ derived from heterogeneous nuclear ribonucleoproteins such as FUS as triggering factors for LLPS.^11–15^

Inspired by the observation that the combination of two or more LCDs may lead to hydrogel formation, and that kinked β-sheets feature less tight interactions compared to steric zippers, the present study exploited low-complexity aromatic-rich kinked segments (LARKS)^10^ to define reversible stimuli-responsive hydrogels.^16,17^

Hydrogels generally represent platforms with peculiar physical and mechanical properties.^18,19^ At a supramolecular level, they are formed by interacting structures composing a network able to entrap water or biological fluids, and their water compartments may be used to host hydrophilic molecules, including Active Pharmaceutical Ingredients (APIs), to serve as reservoirs for controlled release.^20,21^ It has been also demonstrated that peptide-based hydrogels can noncovalently encapsulate low-solubility drugs.^22^ Moreover, they present a network structure resembling the extracellular matrix, which, in conjunction with their inherent flexibility, biocompatibility, and swelling capacities, make them ideal materials for tissue engineering and wound healing.^23–27^ Stimuli-responsive hydrogels, which undergo structural or mechanical changes in response to specific triggers such as temperature,^28^ light,^29^ or pH,^30^ are widely studied as drug delivery materials, as their tunable architecture may help the targeted release of the molecules they carry. Because of the flexible nature of the interactions mediated by LCD, hydrogels composed of these sequences are expected to exhibit many advantages over conventional or stimuli-responsive peptide-based hydrogels.^31^ For example, these hydrogels can function as soft scaffolds that can be extruded through syringe needles and act as injectable matrices and fillers.^32,33^ Moreover, in application of intracellular drug delivery, LLPS-based materials can facilitate translocation across the cell membrane through more efficient and energy-independent mechanisms such as direct cytosolic delivery.^34,35^

Starting from all these premises and to develop new temperature-sensitive biomaterials and unravel correlations between their microscopic features and macroscopic behavior, this work describes the synthesis and the multiscale characterization of the self-assembly of a 26-residue peptide composed of three LARK sequences, designated as L1-L2-L3.^10^

By designing and characterizing additional shorter multi-LARK sequences, which contain only two of the three parental domains (L1-L2, L1-L3, and L2-L3) (Figure 1), we examined the relationship between self-assembling propensity, peptide length, and LCD sequences. Our findings provided the basis for rational design of point mutations capable of modulating the mechanical properties and the temperature-dependent stability of LARKS-based hydrogels, thereby generating new stimuli-responsive matrices for potential biomedical applications like controlled drug-delivery. In particular, the gap that we address here is how the nanoscopic properties defined by peptide sequences, local structural properties of the LARKS assemblies influence the tendency to form LLPS and correlated these properties with the ability to form stimuli responsive hydrogels.

**Figure 1:**
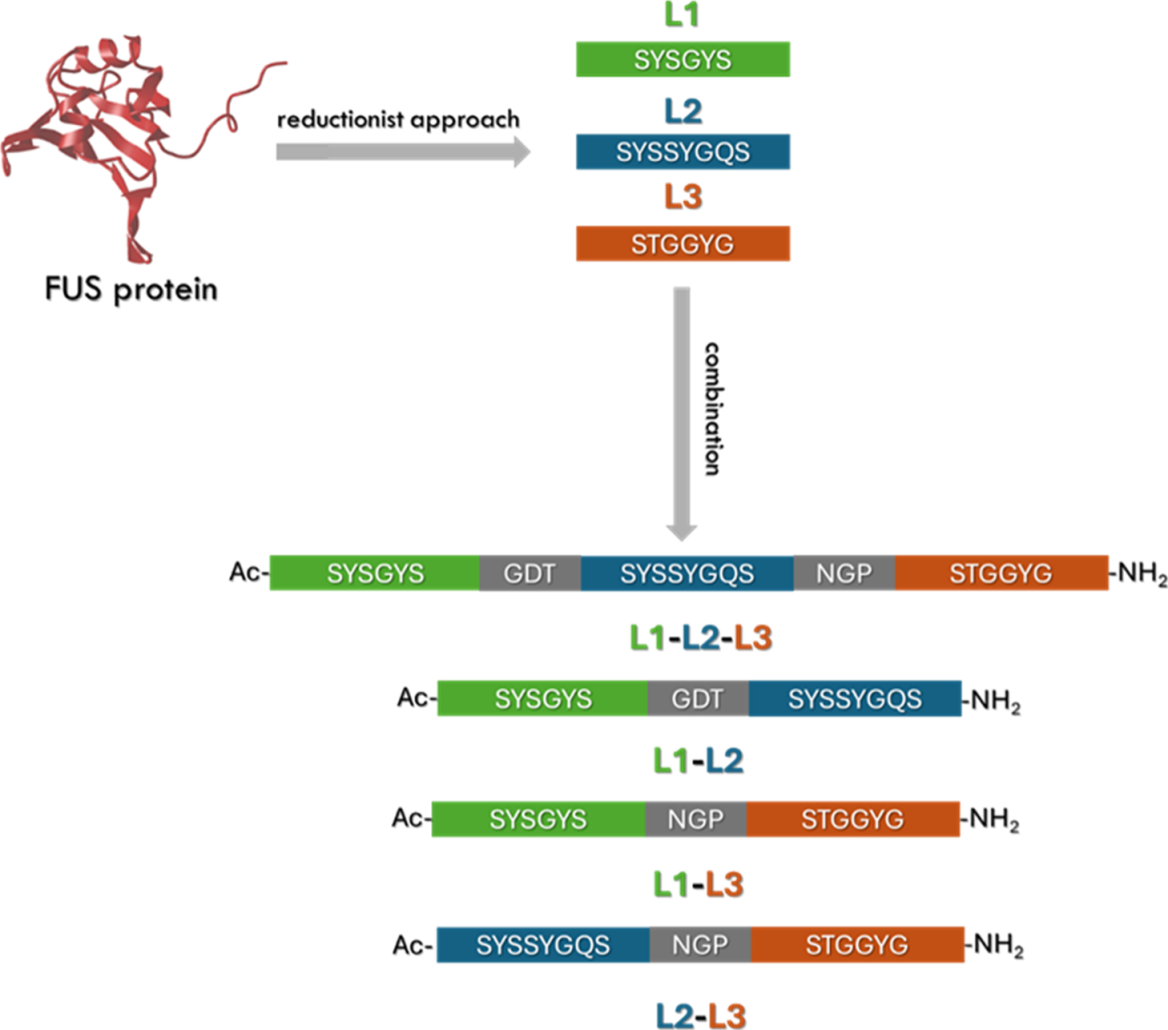
Peptide sequences. Peptide sequences of L1-L2-L3, L1-L2, L1-L3, and L2-L3, reported by one code letter. Each LARK is indicated by a different color (L1 in green, L2 in blue, and L3 in orange), whereas linkers used to connect peptides are depicted in gray.

## Materials and Methods

### Chemicals

Protected N^α^-Fmoc-amino acid derivatives, Rink amide MBHA (4-methylbenzhydrylamine) resin, and coupling reagents commercially available from Calbiochem-Novabiochem (Laufelfingen, Switzerland) were used. All other chemical products were purchased from Fluka (Bucks, Switzerland), Merck (Milan, Italy), or Labscan (Stillorgan, Dublin, Ireland) and they were used as delivered, unless stated otherwise. PBS (Phosphate Buffered Saline) was prepared containing 10 mM phosphate (pH 7.4 ± 0.1).

### Solid-Phase Peptide Synthesis

All the peptide sequences were obtained through standard SPPS (solid-phase peptide synthesis) using the Fmoc/*t*Bu strategy through a Liberty Blue 2.0 microwave peptide synthesizer (CEM, Matthews, NC, USA). The Rink amide MBHA resin, with a substitution rate of 0.65 mmol/g, was used as solid-phase support. Each peptide sequence was synthesized in *N*,*N*-dimethylformamide (DMF) using a scale of 0.2 mmol. Each Fmoc protecting group was removed by treatment with a 10% (v/v) piperidine in DMF for 1 min at 90°. A 2-fold molar excess of the Fmoc-protected amino acid was used for each coupling, using as activating reagents ethyl 2-cyano-2-(hydroxyimino) acetate (oxyma) (1 M in DMF) and *N*,*N*′-diisopropylcarbodiimide (DIC) (1 M in DMF). Each coupling was performed for 2 min at 90 °C. The N-terminus of each sequence was acetylated with a solution of pyridine/acetic anhydride (4/4.7 v/v) in DMF (each treatment for 10 min) in two cycles of treatment. At the end of the procedure, the resin was dried in diethyl ether, and the crude peptides were fully cleaved by treating the peptidyl-resin with a TFA (trifluoroacetic acid)/TIS (triisopropylsilane)/H_2_O (92.5/5/2.5 v/v/v) mixture for 3 h at room temperature. Cold ether was used to precipitate the peptides, which were freeze-dried three times. Pure peptides were obtained via RP-HPLC chromatography with an Agilent 1260 Infinity II Manual Preparative LC System (Agilent, Santa Clara, CA, USA) using a Phenomenex (Torrance, CA, USA) C18 column. The flow rate was set at 20 mL/min and the elution solvents were H_2_O/0.1% TFA (A) and CH3CN/0.1% TFA (B), with (B) increasing from 15 to 70% over 25 min. Analytical RP-HPLC analysis was used to assess the purity of peptides using an Agilent 1260 Infinity II LC system (Agilent, Santa Clara, CA, USA). Peptides were eluted from a Phenomenex C18 column (Torrance, CA, USA) using (A) and (B) as solvents and a method providing the increase of (B) percentage from 5 to 70% over 20 min at a flow rate of 1 mL/min. The identity of peptides was confirmed by MS spectrometry performed by using a LTQ XL Linear Ion Trap Mass Spectrometer, source ESI.

### Sample Preparation

Peptide solutions were dissolved in PBS at concentrations varying from 2.0 μM to 2.0 mM. To allow a complete dissolution, samples were sonicated for 15 min. Hydrogels were prepared by adding PBS to the peptide powder to a final concentration of 2 or 3 wt %. After sonication, samples were stored at 4 °C overnight and the hydrogel formation was macroscopically evaluated through inverted test tube analyses.

### Circular Dichroism (CD) Spectroscopy

Peptide solutions at 2.0 μM and 2.0 mM were placed in 0.1 quartz cell and Far-UV CD spectra were collected through a Jasco J-810 spectropolarimeter equipped with a NesLab RTE111 thermal controller unit at 25 °C. Spectra were recorded from 280 to 190 nm. The following parameters were used as experimental settings for the measures: 0.5 nm step, 1 nm bandwidth, and 1 s collection time per step. For each sample, three scans were collected, averaged, and corrected for the blank to obtain the final spectrum. The spectra in optical density were converted into mean residue ellipticity (Θ, expressed as deg cm^2^ decimol^–1^) by using the following formula:

[Θ] = millidegrees/(path length in millimeters × concentration in M × number of peptide bonds).

### Fourier Transform Infrared (FTIR) Spectroscopy

FTIR spectra for both the liquid and dried samples were collected using a Thermo-Scientific Nicolet iS5 instrument with a DTGS detector JASCO FT/IR4100 spectrometer (Easton MD). Peptide solutions at 2.0 mM were placed in a Specac Pearl liquid cell with CaF_2_ plates. For data acquisition on the dried samples, liquid solutions were left to dry on the cells before the experiment was carried out. Each sample was subjected to a total of 128 scans. Spectra were collected over the wavenumber range 900–4000 cm^–1^ (sensitivity of 1.0 cm^–1^).

### Fluorescence Spectroscopy

A Shimadzu RF-6000 (Kyoto, Japan) spectrofluorometer was used to perform fluorescence measurements. Samples were placed in a 10.0 mm × 5.00 mm quartz cell and the settings used for the measurement were excitation and emission bandwidths of 2.5 nm and temperature of 20 °C. The critical aggregation concentration (CAC) of the peptides was estimated through the evaluation of the self-fluorescence behavior of the peptides. After excitation at 310 nm, the intensity of the emission band at 407 or 417 nm was checked and plotted as a function of concentration. Spectra were also recorded in excitation mode.

Moreover, the ability of the peptides to form β-sheet structures was checked by using Thioflavin T (ThT), a cationic benzothiazole dye showing enhanced fluorescence around 485 nm upon binding to amyloid fibers.^36^ The peptide powder was dissolved at 0.50 mM, 1.0 mM, and 2.0 mM concentrations in PBS, with a ThT concentration of 50 μM. Emission spectra were recorded between 460 and 600 nm (λ_ex_ = 450 nm).

Aggregation kinetics of peptide samples at 2.0 mM were followed by using a FLUOstar Omega (BMG LABTECH) microplate reader. Aliquots of 100 μL of each sample were placed in wells of a 96-well plate and covered with an aluminum foil to avoid evaporation of the solvent. Experimental settings were: λ_ex_ = 355 nm, gain 1000, bottom optic, orbital shake 200 rpm between readings. Emission intensity at 460 nm was recorded over 300 h and subtracted for the blank. Experiments were conducted in triplicate.

### CR Staining and Polarized Optical Microscopy (POM)

30 μL of each peptide solution (at a concentration of 2.0 mM) was placed on a microscope slide and air-dried before being stained with a saturated Congo Red aqueous solution. The sample was then left to dry again and then it was observed under bright-field illumination and between crossed polars by using an OptechMB80-Pol microscope.

### Fluorescence Microscopy

After drop-casting on a microscope slide, 2.0 mM peptide solutions were left to dry overnight. Peptide aggregates in the solid state were analyzed using a Fluorescence OptechMB80-Pol microscope.

### Rheological Studies

Rheological measurements of freshly preformed hydrogels (1000 μL) were performed with a rotational controlled-stress Haake Mars 40 rheometer (Thermo Fisher, Germany) by using a 3.5 cm diameter flat-plate geometry (P35/TI) with a gap of 0.5 mm. The analyses were conducted at different temperatures increasing from 4 to 80 °C and then decreasing from 80 to 4 °C. Amplitude (γ) was set at 0.1% and Frequency 1 Hz. *G*′ (storage modulus) and *G*″ (loss modulus) are reported as a function of the temperature.

### Scanning Electron Microscopy (SEM)

Morphological analysis of xerogels was carried out by field-emission SEM (Phenom ProX, Thermo Fisher Scientific, Waltham, Massachusetts, US). Samples were prepared by drop-casting and air drying 10 μL of hydrogel on an aluminum stub. A thin coating of gold was sputtered using a LUXOR^au^ SEM coated (Aptco Technologies, Nazareth, Belgium) at a current of 25 mA for 75 s. The sputter-coated samples were then introduced into the specimen chamber and images were acquired at an accelerating voltage of 10 kV, through the Secondary Electron Detector (SED).

### Small-Angle X-Ray Scattering (SAXS)

The SAXS scattering experiments were carried out on beamline B21 at Diamond (Didcot, UK). The sample solutions were placed into the 96-well plate of an EMBL BioSAXS robot and then injected via an automated sample exchanger into a quartz capillary (1.8 mm internal diameter) in the X-ray beam. To avoid parasitic scattering, the quartz capillary was kept inside a vacuum chamber. After the sample was injected into the capillary and reached the X-ray beam, the flow was stopped during the SAXS data acquisition. Beamline B21 operates with a fixed camera length (3.9 m) and fixed energy (12.4 keV). A PILATUS 2 M detector was used to record SAXS patterns, and data were processed using dedicated beamline software ScÅtter.

### Fluorescence Recovery after Photobleaching

Fluorescence recovery after photobleaching (FRAP) measurements were acquired using our established protocol at a constant temperature of 37 °C using a Zeiss microscope LSM 980 with an Airyscan 2.^37^ L1-L2 and L2-L3 samples at 1 mM concentration were prepared using a 1:100 molar ratio of FITC-labeled peptides (FITC-L1-L2 and FITC-L2-L3, respectively), obtained by addition, through standard SPPS procedures, of β-Ala and fluorescein isothiocyanate (FITC-NCS), at the N-terminus of each peptide. Solutions were prepared in 1 × PBS buffer, 0.5 mM ammonium sulfate and 10% (v/v) 2-methyl-2,4-pentanediol (MPD). Each FRAP assay consisted of 3 prebleach frames and nine bleach frames, with fluorescence recovery monitored for 12 min at a rate of one frame every 30 s. FRAP experiments were carried out in triplicate, and the time-dependent recovery of fluorescence intensity (%) in the bleached region was reported as a function of time.

### MD Simulations

The stability of the L1 peptide (SYSGYS) in the assembled structure, as resolved by Eisenberg and co-workers,^10^ was evaluated using MD simulations, as previously employed for other similar amyloid-based assemblies.^38^ The starting coordinates were derived from the crystal structure of the self-assembled L1 (PDB code 6BWZ). As a starting model, we considered an assembly composed of a pair of β-sheets of 22 strands (Figure S15a). Both N- and C-termini of the models were kept uncharged during the simulations.

MD simulations were performed with the GROMACS package by using the CHARMM 27 ^39^ force field and a TIP3P^40^ water model. Three simulations were carried out, each extended for 1 ms, for the three systems (L1, L1* and L2**) using periodic boundary conditions in explicit waters (12347 for L1, 12384 for L1* and 12739 for L1**). Bond lengths were constrained by the LINCS algorithm.^41^ The simulations were carried out using the Part Mesh Ewald method^42^ with a grid size of 1.2 Å. A nonbonded cutoff of 14 Å has been selected for the van der Waals. Systems were simulated in *NPT* ensemble by keeping constant temperature (300 K) and pressure (1 atm); a weak coupling to external heat and pressure bath was applied with relaxation time of 0.1 and 0.5 ps, respectively. The integration time step was set to 0.002 ps and the coordinates were saved every 5 ps.

### Doxorubicin Encapsulation and Release

Doxorubicin (Dox) was encapsulated in 3 wt % L1**-L2 hydrogels by dissolving 9 mg of the peptide powder in 300 μL of a 4.5 mM Dox solution in PBS. Analogously to empty matrices, hydrogelation was triggered by leaving the sample overnight at 4 °C. Release kinetics were followed using hydrogels incubated at 25 and 40 °C by adding 600 μL of PBS on top of the matrix. At each time point (0, 0.25, 0.5, 1, 2, 4, 8, 24, 32, 48, 56, and 72 h), an aliquot of 300 μL of the PBS medium was removed and substituted with 300 μL of fresh PBS. The amount of Dox in each fraction was estimated through UV–vis experiments by checking Dox absorbance at 484 nm. Release kinetics were fitted by using the Higuchi model.

## Results and Discussion

### Design and Synthesis of LARK Sequences

Phase-separating peptides were designed by combining multiple LARK sequences and their self-assembly properties were investigated in aqueous environment, including the characteristics of the resulting hydrogels. Three LARKS were selected from the FUS sequence, namely L1 (residues 37–42), L2 (residues 54–61) and L3 (residues 77–82). A first multi-LARK peptide was made from the combination of the three LCDs linked with GDT and NGP sequences, resulting in the Ac-SYSGYSGDTSYSSYGQSNGPSTGGYG-NH2 peptide (L1-L2-L3) as previously described.^10^ Additionally, combinations of two LARKS, namely the L1-L2, L2-L3, and L1-L3 were studied for their aggregative behavior (Figure 1). The same linkers of the full-length sequence were preserved between L1/L2 and L2/L3 domains in our design of the two-LARKS containing peptides to maintain consistency and allow direct comparison with the previously reported system.^10^ The linker between L1-L3 (NGP) was chosen arbitrarily, starting from the premise that the assembly is driven by LARKS fragments and not by linkers, as supported by the findings and conclusions of the earlier study.^10^

All the peptides were synthesized through SPPS on a Rink-amide resin, providing amidated C-terminus, and the N-terminus was acetylated with the aim of avoiding the formation of zwitterions. After the synthesis, all the peptides were purified by preparative RP-HPLC chromatography, and their identity and purity were assessed by analytical RP-HPLC and LC–MS, respectively (Figures S1–S4). Retention time and molecular weight experimentally determined for each peptide are reported in Table S1.

### Self-Fluorescence Studies

The ability of the multi-LARKS to self-assemble was evaluated in physiological buffer conditions (10 mM PBS, pH 7.4). Critical aggregation concentrations (CACs), designated as the minimum concentrations at which they spontaneously self-assemble, were assessed in the absence of any external fluorescence probe and by detecting the nonaromatic intrinsic self-fluorescence at increasing peptide concentrations.^43^ While self-fluorescence is generally associated with amyloid-like aggregates,^44,45^ previous studies demonstrated that it can also be observed as a result of noncanonical amyloid self-assembly. All the multi-LARK sequences here studied presented a near-UV emission, with a maximum of excitation at 310 nm. Upon aggregation, all four peptides showed aggregation-induced fluorescence, with an emission peak centered at 417 nm (Figure S5). Only in the case of L1-L2, a blue shift to 400 nm was observed in the emission when compared to the other constructs (Figure S5).

By plotting the fluorescence intensity at the maximum emission wavelength as a function of concentration, CAC values, calculated at the break point, resulted 128 μM, 236 μM, 153 μM and 175 μM for L1-L2-L3, L1-L2, L1-L3, and L2-L3, respectively (see Table S2). These findings indicate that the reduction from three to two LARKS has no significant effect on the concentration at which the peptides aggregate and start to display photoluminescence.

We also observed that the aggregated multi-LARK peptides maintain their capability to self-fluoresce in the solid state (Figure 2). In particular, samples were prepared by drop-casting peptides from a solution in phosphate buffer at a concentration of 2.0 mM, and the resulting films were imaged by bright field and fluorescence microscopy in various spectral regions. All the samples treated in this way displayed high fluorescence in the green region of the visible spectrum, whereas only L1-L2, L1-L3 and L1-L2-L3 showed a particularly evident emission in the blue field. Interestingly, in contrast to what observed in the liquid state, all the samples showed a weak emission in the red when analyzed in the solid state. Moreover, of the four multi-LARK peptides, L2-L3 appeared to adopt a peculiar, aggregated state, with resulting objects smaller than the other cases. The lower emission exhibited by this di-LARK construct in both the blue and the green fields can be attributed to the smaller dimensions of the aggregates. It should be noted, however, that because the physical mechanism underlying fluorescence emission of these aggregates in the near-UV and visible regions remain a matter of debate,^45^ it is challenging to attribute the observed variations in emission to a specific structural factor. The phenomenological observation of this fluorescence variability is nevertheless valuable, as it could serve as a benchmark for future studies aimed at understanding the chemical and physical basis of this puzzling process.

**Figure 2:**
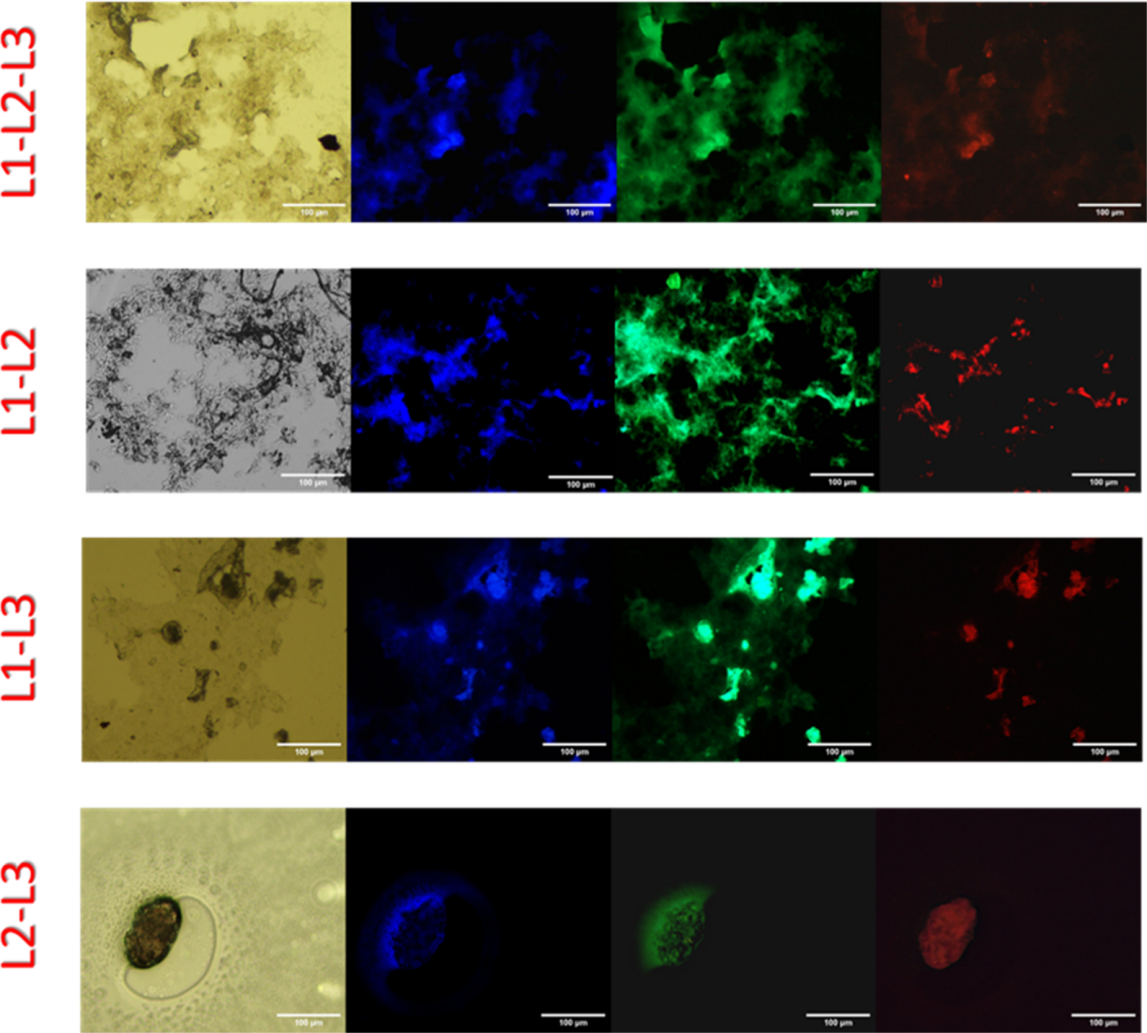
Fluorescence properties of peptides at the solid state. Fluorescence microscopy of L1-L2-L3, L1-L2, L1-L3 and L2-L3 peptide aggregates: bright field images (left) and fluorescence (right three images) excited in the spectral regions of GFP (λ_exc_ = 488 nm, λ_em_ = 507 nm), DAPI (λ_exc_ = 359 nm, λ_em_ = 461 nm), and rhodamine (λ_exc_ = 359 nm, λ_em_ = 461 nm) filters (scale bar = 100 μm).

### Secondary Structure Characterization

The secondary structure of the peptides was characterized using Circular Dichroism (CD) and Fourier Transform Infrared (FTIR) spectroscopy.^46,47^ CD studies were conducted on peptide solutions at concentrations below and above the CAC (see Table S2).

For all the samples we observed random-coil conformations when the peptides were at a concentration (C) of 2.0 μM (C < CAC), as shown by a negative band between 195 and 205 nm ascribable to π–π* transitions of the amide bond (Figure S6). In contrast, at a concentration of 2.0 mM (C > CAC) the conformations of the multi-LARKS were dominated by β-sheet conformation, as suggested by the presence of a negative signal in the region of n–π* transitions (Figure 3a). This band resulted to be centered around 220 nm for all the multi-LARK sequences. Additional evidence for a β-sheet organization was substantiated by a positive CD signal around 195–200 nm, as observed in the case of L1-L2-L3, L1-L2, and L2-L3 (Figure 3a). Finally, a positive band at 230 nm was visible in the spectra of L1-L2-L3 and L1-L3 spectra, which is attributable to π -π* transitions of the phenol ring.^48^

**Figure 3:**
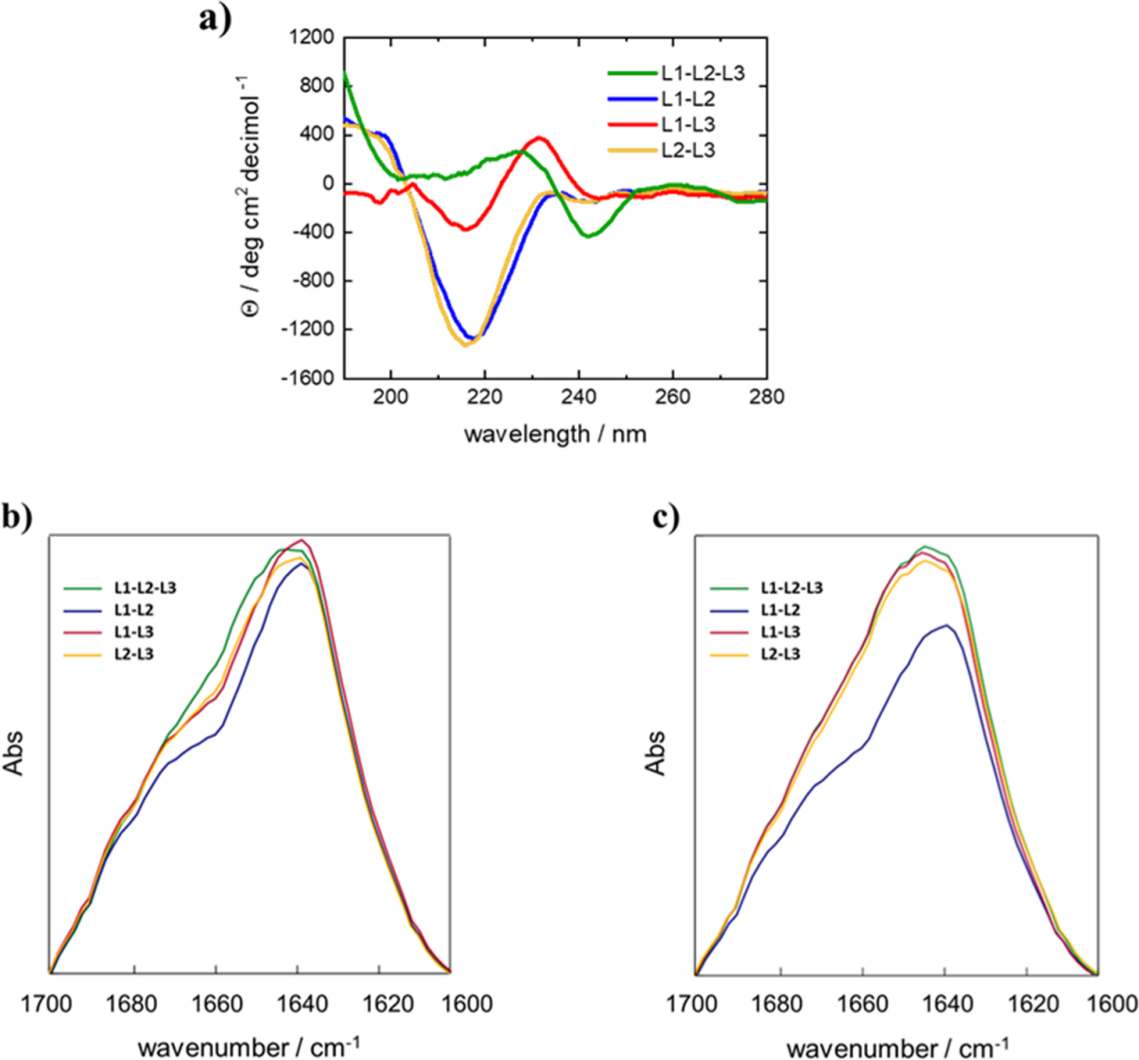
Secondary structure analyses. (a) CD spectra of L1-L2-L3, L1-L2, L1-L3 and L2-L3 at a concentration of 2.0·mM, higher than the CAC one. FT-IR spectra of L1-L2-L3, L1-L2, L1-L3, and L2-L3 (b) in solution and (c) at the solid state.

FT-IR spectra in the Amide I region (1600–1700 cm^–1^) were used to support data derived from CD experiments. Samples at 2.0 mM concentration were studied both in solution and in the solid state, showing no significant differences in the FTIR spectra measured in PBS suspension or in the dried form. In particular, measurements in both solid and liquid states showed a signal around 1642 cm^–1^ in the spectrum deconvoluted in the amide I region of all the peptides, which is indicative of the presence of β-sheets (Figure 3b,c) thereby confirming the results from CD experiments.

### Colorimetric Assays: Thioflavin T and Congo Red

To further characterize the conformational properties of the self-assembled multi-LARK sequences, fluorescence experiments with the Thioflavin T (ThT) dye, a probe commonly used to monitor amyloid formation, were carried out. Upon binding to amyloid fibrils, ThT generates strong fluorescence at 482 nm when excited at 450 nm.^36,49^ The results collected from our experiments indicated that LARKS-derived aggregates are ThT positive, a characteristic generally associated with amyloid-like assemblies. Indeed, for high peptide concentrations, the emission peak at 482 nm was observed in the liquid state for samples prepared in the presence of 50 μM ThT (λ_ex_ = 450 nm, Figure S7). In addition, our data suggests that ThT interferes to some extent with the assembly process of the LARK-sequences, as images collected with both the bright field and the GFP filter revealed aggregates of considerably smaller size to those obtained in the absence of ThT (Figure 2). Consistently with self-fluorescence measurements, the L2-L3 construct showed smaller aggregates and weaker ThT fluorescence intensities than L1-L2-L3, L1-L3, and L1-L3. The peculiar self-assembly behavior of L2-L3, as observed with multiple orthogonal measurements, provides evidence that the L1 sequence, rather than the peptide length, is the key promoter of the aggregation of the multi-LARK peptides.

Finally, to further elucidate the conformational properties of the aggregates of the multi-LARK constructs, Congo Red (CR) experiments^50^ were carried out on dried samples. CR generates dichroic and birefringent effects under cross-polarized light as a consequence of the staining via both hydrophobic and electrostatic interactions. Samples prepared on glass slides were stained, after the drying process, with a saturated CR solution and analyzed with the microscope under cross-polarized light (Figure S8). A weak apple-green birefringence was detected for all the multi-LARK samples, thus assessing not only the presence of β-sheets in the aggregates but also confirming the amyloid-like nature of these aggregates.^51^ Consistent with previous different measurements, aggregates of L2-L3 were again found to be smaller and less responsive to the CR staining.

### Kinetics of Self-Assembly of the Multi-LARK Sequences

The aggregation kinetics of the four multi-LARK sequences were monitored using fluorescence experiments, by exploiting the photoemissive behavior of the peptides in the blue field. As the fluorescence emission of these sequences require the aggregation state, as shown by the analyses conducted in solution, it represents a convenient probe of the self-assembly process. Upon excitation at 355 nm of 2.0 mM peptide solutions, the emission at 460 nm was recorded across a 300 h period (Figure S9). By reporting the fluorescence intensity as a function of time, we observed consistent trends across the four multi-LARKS samples, characterized by three different kinetic phases. In the initial part of the aggregation, corresponding to the first ∼50 h for L1-L2-L3 and L1-L3, and the first ∼85 h for L1-L2 and L2-L3, the kinetic measurements showed constant fluorescence intensity values. This lag-phase was followed by a linear increase of the fluorescence signal at 460 nm in a relatively short period for all the samples (between 5 and 30 h) except in the case of L1-L2, where this second phase was longer (∼80 h). In the third phase of the kinetic profile, the fluorescence intensity was observed to adopt hyperbolic growth until the end of the measurement. This phase was not attributable to sample precipitation, which was avoided using agitation, or to an increase in peptide concentration, as solvent evaporation was prevented by sealing the plate with aluminum foil. In fact, at the end of the experiment, the recovered samples resulted unchanged in both appearance and volume. As a result, the changes in the emission behavior were attributed to a reorganization of the supramolecular aggregates. The rearrangements did not involve alterations in the secondary structure of the aggregates, as CD measurements carried out on L2-L3 samples at three different time points of the kinetic profile (24 h, 120 and 264 h) generated completely superimposable spectra (Figure S10a). In agreement with the single fluorescence emission of 460 nm (λ_ex_ = 350 nm), the intensity of fluorescence spectra in both emission and excitation were found to consistently increase over time (24 h, 120 h and 264 h, Figure S10b).

### Hydrogel Formulation

In the study previously reported by Eisenberg and co-workers,^8^ the L1-L2-L3 sequence was shown to form hydrogels when dissolved in water at a concentration of 5 wt % after an overnight incubation at 4 °C. Consequently, we investigated whether the multi-LARK sequences composed of two LCDs exhibit similar self-assembly properties. Self-supporting hydrogels of L1-L2 and of L1-L2-L3 were obtained starting from a concentration of 2 wt % when 10 mM PBS was used in place of water (inverted test tube, Figure 4a). The effect of the salts (NaCl, KCl, and phosphate buffer components) in helping the hydrogel aggregation process at lower concentrations was previously demonstrated for other peptide formulations.^52^ From a biomedical perspective, achieving gelation at lower peptide concentrations is highly desirable to minimize potential cytotoxicity and facilitate in vivo applications. In contrast, no gel formation was observed for the L1-L3 and L2-L3 sequences, both containing the L3 LARKS. This sequence, in comparison to the L1 and L2 motifs, features a higher content of glycine residues and a single tyrosine, while L1 and L2 each contain two. The different behavior of L3 might therefore arise from a reduced number of π–π stacking interactions, a known driving factor in the gelation process, or the higher entropic cost associated with glycine residues. Moreover, L1-L2-L3 and L1-L2 peptides present slightly higher CAC values (Table S2), suggesting higher water affinity than the two analogs that are unable to gel.

**Figure 4:**
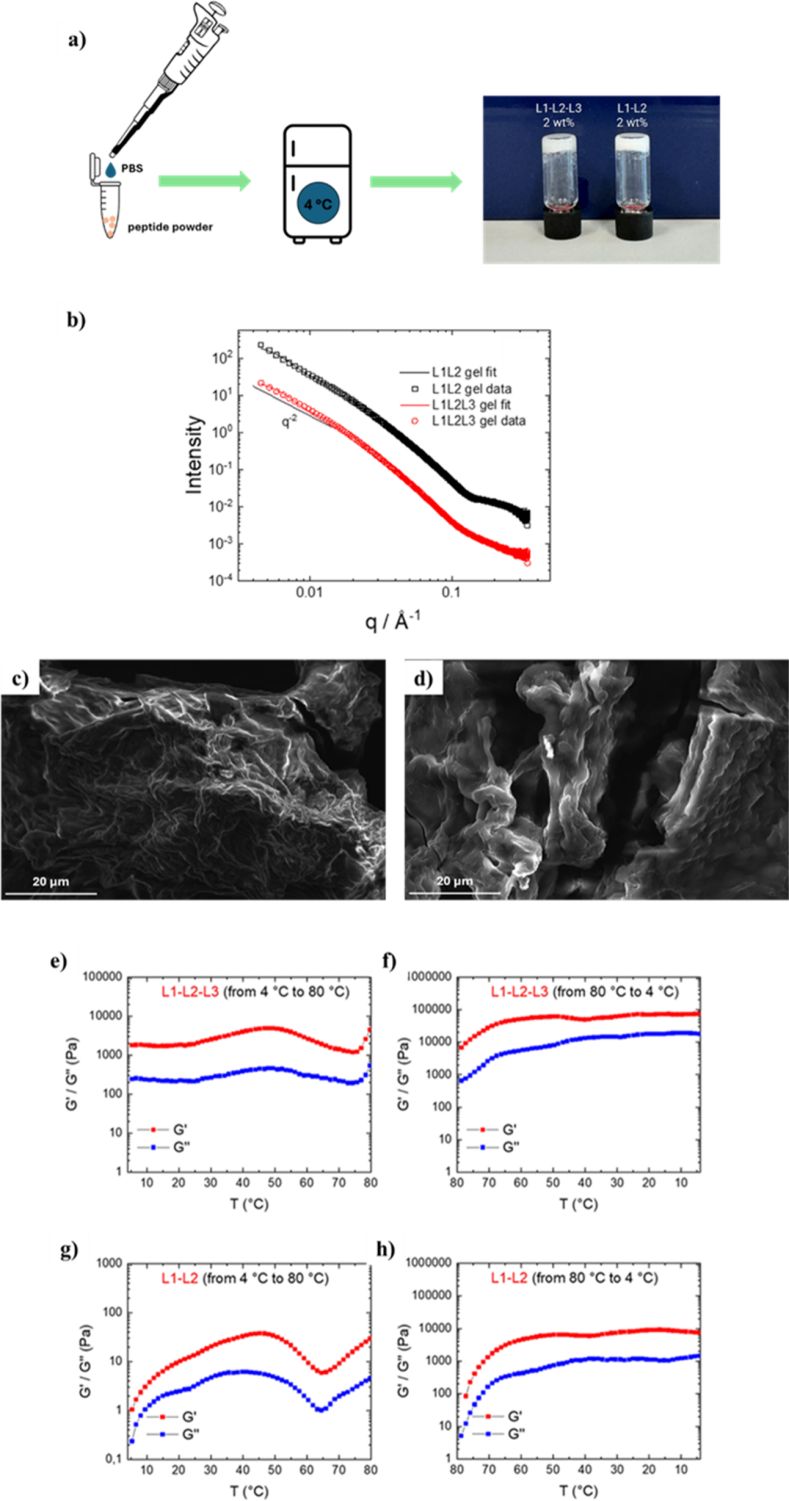
Hydrogel formulation and characterization. (a) Schematic representation of hydrogel formulation procedure: the peptide powder is dissolved in PBS at 2 wt % concentration and stored at 4 °C overnight. Hydrogel formation for L1-L2-L3 and L1-L2 is demonstrated by inverted test tube analyses. (b) SAXS data and model form factor fits (parameters in Table S3) of hydrogels. The open symbols are measured data (for clarity, every fifth data point is plotted) and the lines are the model fits. The data for L1-L2-L3 are divided by *a* factor of 10 for ease of visualization. Selected SEM micrographs of peptide hydrogels at a concentration of 2 wt %: (c) L1-L2-L3, (d) L1-L2. Magnification and scale bar are 6300× and 20 μm, respectively. Rheological analyses as a function of the temperature for L1-L2-L3 and L1-L2 hydrogels at 2 wt % concentration. (e) *G*′ (storage modulus) reported as a function of the temperature increasing from 4 to 80 °C for L1-L2-L3; (f) *G*′ (storage modulus) reported as a function of the temperature decreasing from 80 to 4 °C for L1-L2-L3; (g) *G*′ (storage modulus) reported as a function of the temperature increasing from 4 to 80 °C for L1-L2; (h) *G*′ (storage modulus) reported as a function of the temperature decreasing from 80 to 4 °C for L1-L2. Amplitude (γ) was set at 0.1% and Frequency 1 Hz.

### Morphological Insight on Peptide Solutions and Hydrogels (HGs)

Further structural information on the self-assembly of the multi-LARK peptides in the solution state and in the gel phase was obtained using small-angle X-ray scattering (SAXS) and scanning electron microscopy (SEM) techniques. Data measured with samples in 10 mM PBS (Figure S11) present a characteristic scaling of intensity with wavenumber *I*–*q* ^–2^ approximately, indicating the presence of planar structures (nanosheets or nanotapes).^53^ The data could be fitted using a form factor that describes the density profile across planar structures represented by three Gaussian functions, one representing the electron-depleted interior and the other two (identical functions) representing the electron-rich surfaces (Table S3 for the fitting parameters).^54–56^

The same model was used to fit the data for 2 wt % hydrogels (Figure 4b). The resulting fits showed very good quality, particularly for the hydrogels, where the maxima of the form factor at high q values become more pronounced due to the planar structures. The morphology of the multi-LARKS aggregates was also studied using SEM (Figure S12). The resulting images showed elongated tubular clusters for L1-L2-L3 and L2-L3 aggregates, resulting in holes, whereas the triple-LARK peptide displayed a more rounded surface.

In comparison, the L2-L3 aggregates were found to adopt a cubic morphology (Figure S12d), while the L1-L2 sample displayed a “folded” morphology (Figure S12b), which preserved a tubular interconnecting organization. Peculiar image characteristics were found for L1-L3 (Figure S12c), displaying the formation of films that are partially broken, potentially as a result of the drying process. Finally, while SEM images for the L1-L2 peptide in solution and the corresponding xerogel revealed a very similar morphology (Figure 4d), in the case of L1-L2-L3 the hydrogel state showed a more intricate network of fibers compared to the 2.0 mM solution (Figure S12a).

### Fluorescence Recovery after Photobleaching

FRAP assays were conducted to assess whether the phase-separation propensity of the FUS protein is retained in its derived peptides. Among the four peptide sequences, FRAP experiments were specifically performed on L1-L2 and L2-L3 to compare one sequence that exhibits gelation properties to another that does not. Interestingly, our data identify a correlation between the ability of the peptides to form hydrogels and the dynamic behavior of their phase-separated assemblies in the micrometer scale.^57^ L1-L2, which can form hydrogels, assembled into condensates (perfectly spherical droplet) with an average diameter of ∼5 μm (Figure 5a) and exhibited complete fluorescence recovery in FRAP experiments, indicative of a liquid-like internal environment with high molecular mobility (Figure 5b–d). In contrast, L2-L3, which does not form hydrogels, generated smaller condensates (∼2 μm) (Figure 5e) with no fluorescence recovery (Figure 5f–h) suggesting a more rigid and less dynamic structure. These observations are consistent with electron microscopy analyses showing a reduced aggregation tendency for L2-L3 compared to L1-L2. These findings suggest a link between hydrogel-forming propensity and the ability of the peptides to phase-separate into dynamic, liquid-like condensates.

**Figure 5:**
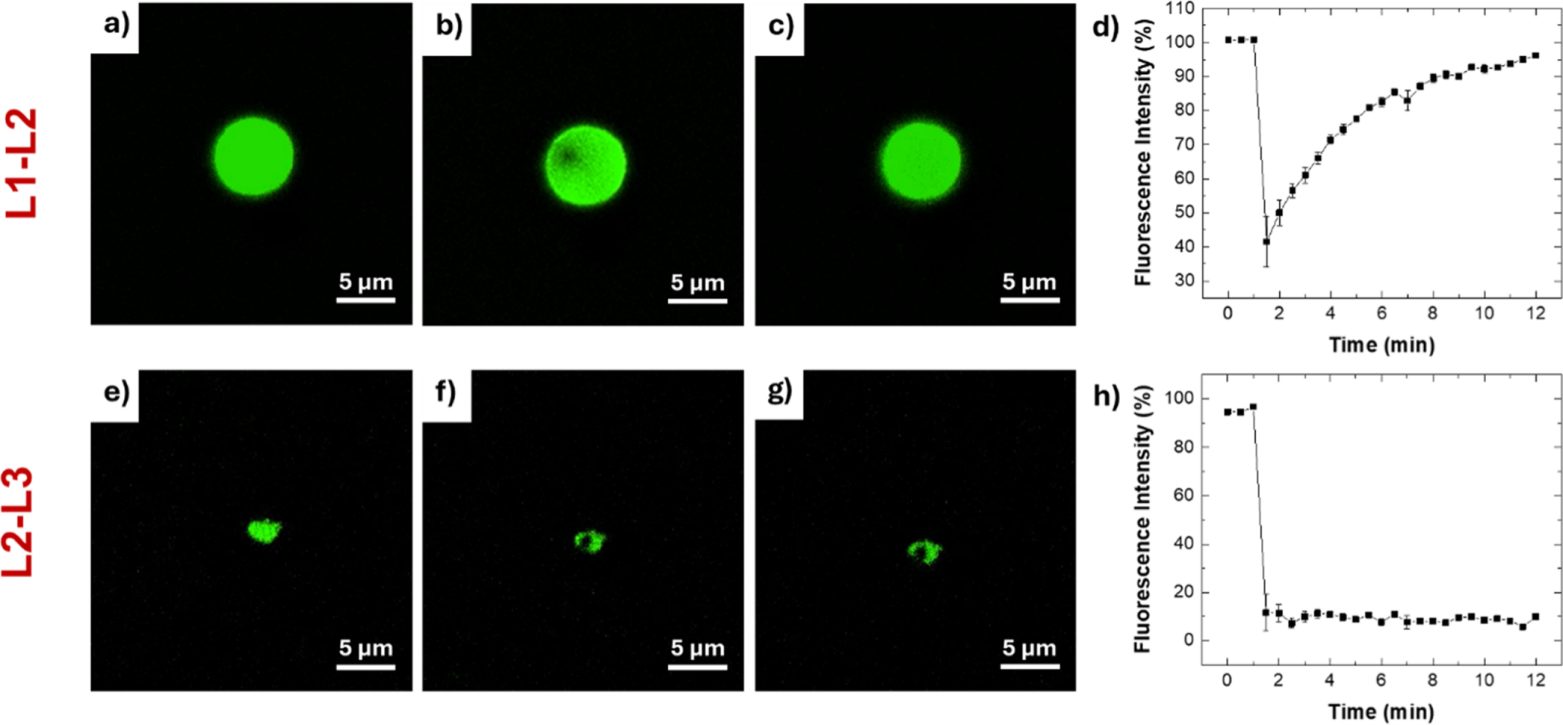
FRAP experiments. Confocal micrographs of FITC-L1-L2/L1-L2 condensates (molar ratio 1:100) measured (a) prior photobleaching (0 min), (b) after photobleaching (1.5 min), and (c) after recovery (12 min); (d) Time-dependent recovery of fluorescence intensity (%) in the ROI of the bleaching, with error bars indicating the standard deviation from three independent replicates. Confocal micrographs of FITC-L2-L3/L2-L3 condensates (molar ratio 1:100) measured (e) prior photobleaching (0 min), (f) after photobleaching (1.5 min), and (g) after recovery (12 min); (h) time-dependent recovery of fluorescence intensity (%) in the bleached region, with error bars indicating the standard deviation from three independent replicates.

### Stimuli-Responsive Rheological Properties

To address the impact of the primary sequence on the hydrogel’s sensitivity to external stimuli, their mechanical behavior as a response to pH and temperature triggers was tested.^58^ Preformed L1-L2 and L1-L2-L3 hydrogels were put in contact with both acidic (pH 5) and basic (pH 10) solutions at room temperature and were macroscopically observed over time up to 7 days. No significant variations were detected, thus indicating high stability of the gels to the acid and basic pH conditions. This behavior can be rationalized considering that the presence of ionizable acidic or basic amino acids typically drives pH-responsive properties in peptide-based systems. In our case, the sequences contain only a single acidic residue (Asp, D), which is located within the linker region rather than in the LARKS domains considered to be responsible for driving the gelation process.

To study the mechanical behavior of the matrices, hydrogels from L1-L2-L3 and L1-L2 sequences were subjected to rheological measurements. The storage (G′) and loss (G″) moduli, two key parameters describing the viscoelastic behavior of materials, were studied as a function of the temperature. Measurements were conducted across the entire temperature range, increasing from 4 to 80 °C and subsequently decreasing from 80 to 4 °C. Interestingly, the G′ consistently showed higher values than the *G*″ at every measurement point during the experiments, demonstrating the stability of the hydrogels across the measured temperature range (Figure 4e–h). However, the heating/cooling process induced significant increase in the rigidity of both samples. In particular, for the L1-L2-L3 sample, G′ was found to be 1967 Pa at 4 °C, increasing to 4708 Pa upon heating to 80 °C and further rising to 75762 Pa when the temperature was decreased back to 4 °C. For the L1-L2 sample, the initial values of G′ at these temperatures were 1 Pa at 4 °C, 32 Pa at 80 °C, and 7314 Pa upon cooling back at 4 °C. To enhance the hydrogel disaggregation response to temperature increase, we adopted a design strategy focused on incorporating disruptive elements into the peptide sequences. This approach stems from the insights that the L3 sequence has the potential to inhibit the aggregation of the multi-LARK peptides. Following this rationale, we initially synthesized a multi-LARK sequence made of L3-L2-L3 LCDs (Figure S13a). In line with the evidence of the key role of L1, this sequence was unable to form hydrogels at concentrations ranging from 2 to 5 wt % (Figure S13b).

In order to further exploit our findings, we rationally designed and characterized mutational variants of L1 to obtain mechanistic evidence of its role in the supramolecular organization of these systems.^55^ Specifically, we aimed at gradually morphing the L1 sequence into L3, initially mutating of the second serine residue into a glycine (L1*-L2 di-LARK peptide reported in Figure S14). To further destabilize the hydrogels based on multi-LARKS containing L1, we further mutated the L1 by substituting the first tyrosine of the sequence into a threonine (designated as L1**), thus effectively depleting a stacking aromatic interaction and H-bonds stabilizing its amyloid-like assembly (PDB code 6BWZ) (Figure 6a).

**Figure 6:**
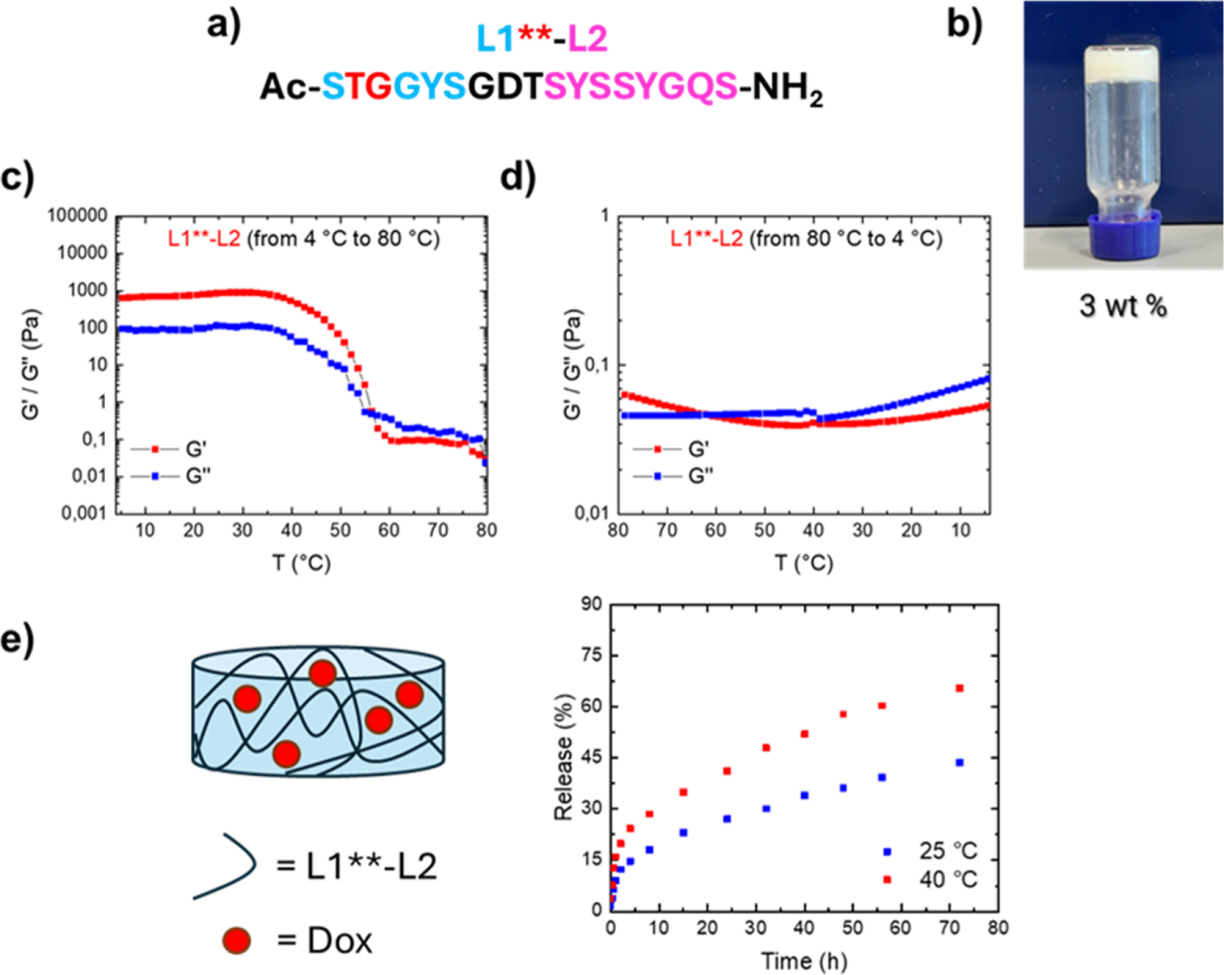
Formulation and characterization of L1**-L2 hydrogel and Dox release kinetics. (a) L1**-L2 sequence; (b) inverted test tube for L1**-L2 hydrogel at 3 wt % concentration; (c) *G*′ (storage modulus) reported as a function of the temperature increasing from 4 to 80 °C; (d) *G*′ (storage modulus) reported as a function of the temperature decreasing from 80 to 4 °C. (e) Release kinetics of Dox from L1**-L2 HG over 72 h at two different temperatures, 25 and 40 °C.

To preliminary assess the effects of these mutations, we studied the nanoscale properties of the L1 assembly and its derived mutants—L1* and L1**—using molecular dynamics (MD) simulations. Our working hypothesis aimed to assess the stability of the smallest fibril-like construct based on the experimental structure of the assembled L1 (6BWZ), modeled as a single steric zipper unit comprising two opposing β-sheets solvated in water (Figure S15a). The simulations were therefore designed to evaluate the contribution of the SYSGYS, SYGGYS and STGGYS motifs to the stability of this fundamental structural unit, which is expected to underlie the formation of higher-order supramolecular architectures. In the simulation time of 1 μs, the L1 construct showed significant stability, with the Cα root-mean-square fluctuation (RMSD) plateauing at 0.28 nm (Figure S15b). As a key element in influencing the Cα RMSD is given by bending events in the elongated fibril, we also evaluated the fraction of preserved native contacts along the simulation, showing that L1 is able to maintain 96.7% of the contacts during the simulations (Figure S16). A different scenario emerged in the case of L1*. The simulation of this construct was associated with an immediate rise of the Cα RMSD to values higher than 3.5 nm. In particular, L1* was able to retain clusters of the initial structure, but fragmented into smaller units (Figure S15c), yielding to a fraction of native contacts of 53% at the end of 1 μs simulation (Figure S16). Finally, the L1** construct was found to be significantly unstable compared to the 6BWZ structure. As in the case of L1*, the Cα RMSD in the simulations of this construct immediately rose to very high values (>3.5 nm, Figure S15a), indicative of structural disruption. In the 1 μs simulation, the fraction of preserved contacts in L1** dropped to 29%, generating assemblies that resembled condensate phases (Figure S15d).

When we tested the mutated sequences experimentally, we found that L1*-L2 di-LARK peptide forms hydrogels at 2 wt % concentration (Figure S14). Rheological analyses on this peptide showed similar trends in G′ compared to other hydrogels previously analyzed, without any clear sign of disruption. In particular, the initial value of G′ at 4 °C was 818 Pa and, differently from the L1-L2-L3 and L1-L2 cases, decreased to 497 Pa by increasing the temperature to 80 °C (Figure S14c), thus showing a slight sensitivity to the heating stimulus. However, upon temperature reduction to 4 °C G′ strongly increased to 154332 Pa (Figure S14d).

On the other hand, di-LARKS of sequence L1**-L2 could form hydrogels only at 3 wt % concentration (Figure 6b), thus confirming that the Tyr → The mutation in the second residue of L1 induces alterations of the hydrogelation capacity, as also suggested by MD simulations. Moreover, rheological analyses revealed that, when the temperature reaches 55 °C, this hydrogel undergoes breakdown and becomes unable to reform upon cooling (Figure 6c,d).

Having found a di-LARK sequence able to act as a temperature-responsive matrix, we tested if L1**-L2 hydrogel can be used for the release of a model drug. In particular, Doxorubicin (Dox), an antitumor antibiotic belonging to the anthracycline family and acting as an inhibitor of topoisomerase II and as a DNA intercalating agent,^59^ was encapsulated at 4.5 mM concentration into L1**-L2 HG and consequently its release from the matrix was determined at 25 and 40 °C. Because of its cardiotoxic nature, Dox encapsulating nanoformulations such as liposomes^60,61^ have been proposed as an alternative strategy for its administration. Recently, peptide-based hydrogels and nanogels have also been tested as Dox reservoirs.^21^ Herein, encapsulation was achieved by dissolving the L1**-L2 peptide powder in the Dox solution and hydrogelation was triggered via overnight incubation at 4 °C. A hydrogel was successfully obtained and, since no syneresis phenomena were detected, an encapsulation ratio of 100% was confirmed. The release of Dox was then evaluated over 72 h at 25 and 40 °C. These respectively represent the control temperature to evaluate passive release from hydrogels and a typical temperature found in pathological environments such as inflamed or tumoral tissues (39–42 °C) (Figure 6e). The estimated amount of Dox release was 43% at 25 °C and 65% at 40 °C, thus demonstrating the suitability of the designed L1**-L2 matrix for applications in controlled drug delivery in inflammatory/tumoral sites, which typically experience higher temperatures than normal tissues. Release kinetics were fitted with the Higuchi equation^62^

$$Q=k_{H}\cdot t^{1/2}$$

where *Q* is the amount of drug released per unit surface area (mg/cm^2^), *k*_H_ is the Higuchi release rate constant (mg/cm^2^·h^1/2^) and *t* is the time in hours.

This equation describes drug release from a homogeneous matrix system where diffusion is the controlling mechanism, and the amount released is proportional to the square root of time. Calculated *k*_H_ are reported in Table S4. At 40 °C, *k*_H_ values are approximately twice those at 25 °C, indicating faster drug release due to increased diffusivity at higher temperature. In both cases, *k*_H_ peaks within the first hour and then gradually decreases, stabilizing after ∼48 h, suggesting an initial burst release followed by diffusion-controlled kinetics.

## Conclusions

Reductionist approaches have significantly advanced peptide supramolecular chemistry, enabling a new level of understanding and control of the phenomena regulating the self-assembly process. Initially applied to the study of amyloid-like peptides,^63^ more recently this strategy has allowed the identification of low complexity domain (LCD) sequences as key elements responsible for the phase-separation activity in proteins such as FUS, resulting in the formation of membrane-less organelles in vivo. The interest in these sequences arises from the possibility of using them as building blocks for the definition of novel biomaterials, where weaker and less regular self-assembly forces may endow the materials with the ability to form and disaggregate in response to external stimuli. The switch in the physical and/or mechanical properties of peptide assemblies, for example as a result of changes in pH, temperature, or magnetic field, may have numerous relevant applications across various biomedical fields. In particular, this approach is expected to promote new strategies for the targeted release of active pharmaceutical molecules in specific tissues, to enhance the definition of sensors with improved sensitivity and specificity, or to enable the generation of self-healing materials. In this study, building on the discovery of LARKS and the elucidation of the underlying principles of their self-assembly, we utilized a combination of LCD-based fragments as building blocks for multi-LARK peptides (L1-L2-L3, L1-L2, L1-L3 and L2-L3), thus establishing the foundation for the design of stimuli-responsive materials based on liquid–liquid phase separations. All these sequences showed the ability to self-assemble into aggregates featuring self-fluorescence in both aqueous suspensions and in the solid state. Self-fluorescence was exploited to estimate their CAC values by monitoring the intensity of the aggregation-induced emission peak at different peptide concentrations. We investigated the conformational bases of the self-assembly of these multi-LARKS and found a common aggregation pathway, driven by β-sheet formation. In addition, the multi-LARKS aggregation resulted in positive responses to ThT and CR assays, indicating that the resulting aggregates exhibit amyloid-like properties. In the solid state, however, some differences were found across the constructs, with peptides containing the L1 sequence generating aggregates with similar morphologies, in contrast to those formed by L2-L3, which aggregate into smaller species. Numerous data of this study indicated that the presence of L1, rather than the peptide length, is the primary factor driving multi-LARKS aggregation at the micrometer scale. At a suitable concentration of 2 wt %, and following overnight storage at 4 °C, L1-L2-L3 and L1-L2 generated self-supporting hydrogels exhibiting resistance to temperature changes. Having identified L1 as a strong promoter of multi-LARKS self-assembly, we designed mutations to make it more similar to L3 when included in multi-LARK peptides. The design and testing generated a thermosensitive hydrogel formed by the L1**-L2 di-LARKS, which showed the ability to be disrupted when the temperature was raised to 55 °C. This result demonstrates that the rational design of new LARKS motifs can lead to the generation of matrices having novel biomaterial properties for biomedical applications. We provide additional support for these conclusions by showing that hydrogels formed by L1**-L2 can act as a drug-reservoir matrix to release with high efficiency a selected model antitumor drug at 40 °C, thereby demonstrating the stimuli-responsivity of the biomaterial and its potential for biotechnological and medical applications.

## Supplementary Material



## Notes

Additional physicochemical and structural characterization of peptides and the corresponding hydrogels (PDF)
