# Supplementary material for: Stimuli-Responsive Hydrogels from Liquid–Liquid Phase Separations of FUS-Derived Peptides
Source: ACS Appl Mater Interfaces. 2025 Sep 24;17(40):55981–93. doi: 10.1021/acsami.5c15249 (PMC12516689; doi:10.1021/acsami.5c15249)
Supplement: Supplementary file 1 [file am5c15249_si_001.pdf]

Supporting Information

Stimuli-responsive Hydrogels from Liquid-Liquid Phase Separations of FUS-derived peptides

Elisabetta Rosa,[†] *Mariantonietta Pizzella,*[†] *Luca Cimmino,*[¥] *Valeria Castelletto,*[‡] *Ian W. Hamley,*[‡] *Luigi Vitagliano,*[§] *Alfonso De Simone,*[†,*] *Antonella Accardo*[†,*]

[†] Department of Pharmacy, Research Centre on Bioactive Peptides (CIRPeB), University of Naples "Federico II", Via De Amicis 95, 80145 Naples, Italy.

[¥]IRCCS SYNLAB SDN, Via Ferraris 144, 80146 Naples, Italy.

[‡]School of Chemistry, Pharmacy and Food Biosciences, University of Reading, Berkshire RG6 6AD, United Kingdom.

[§]Institute of Biostructures and Bioimaging, CNR, Via Castellino 111, 80131 Naples, Italy

*Email address corresponding author:

alfonso.desimone@unina.it; antonella.accardo@unina.it

a)

b)

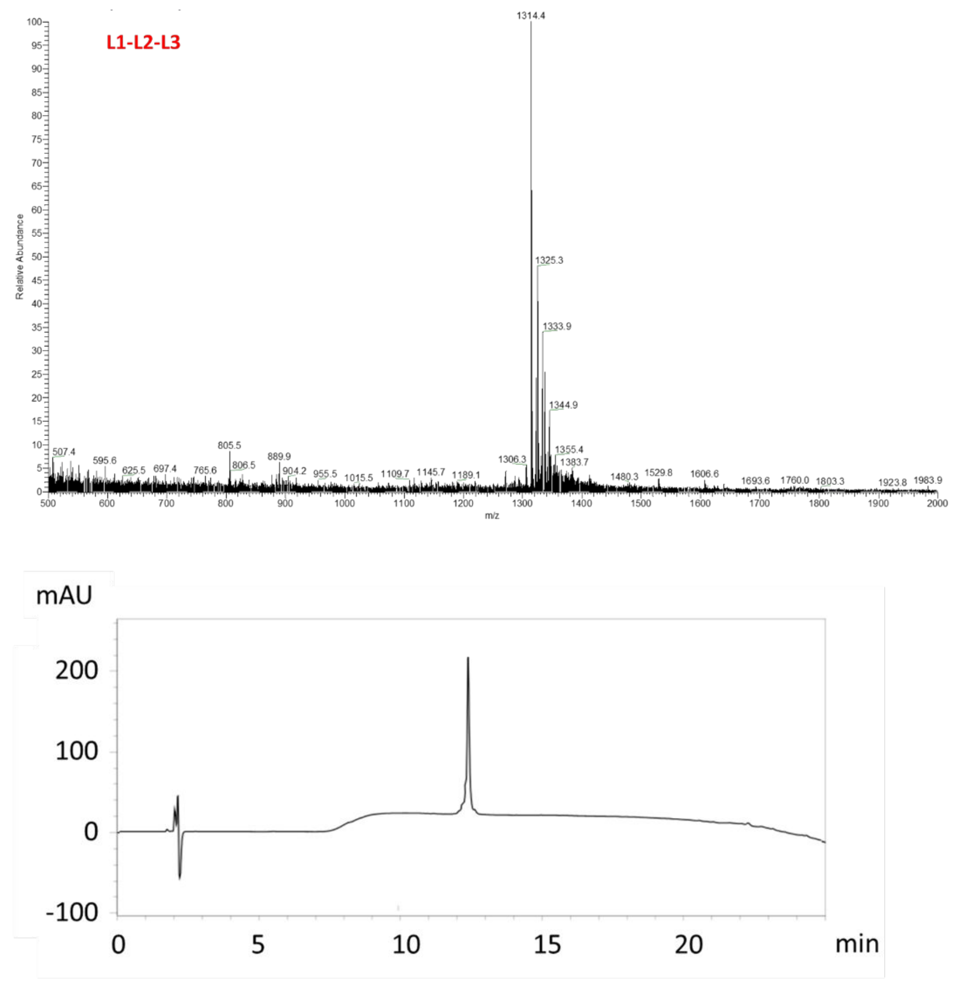

Figure S1: Physicochemical characterization of L1-L2-L3 peptide: a) ESI mass spectrum and b) RP-HPLC chromatography

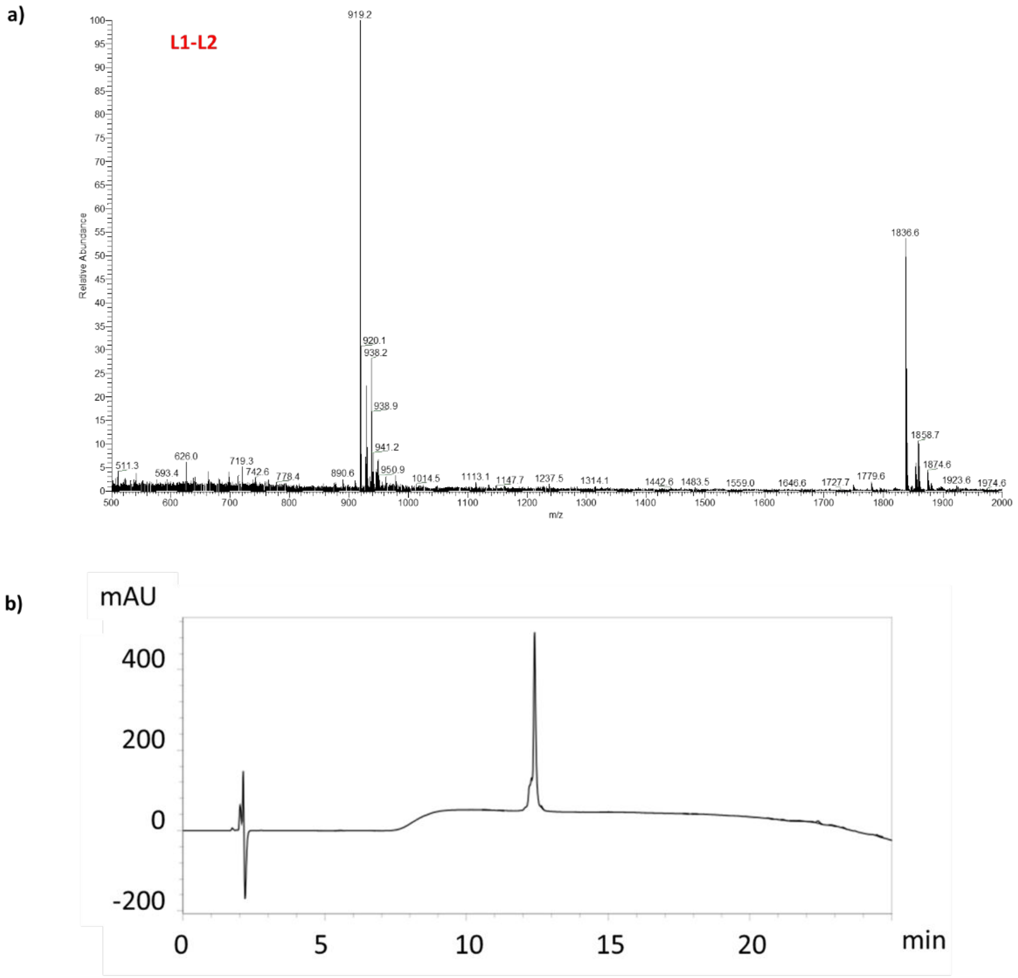

Figure S2: Physicochemical characterization of L1-L2 peptide: a) ESI mass spectrum and b) RP-HPLC chromatography

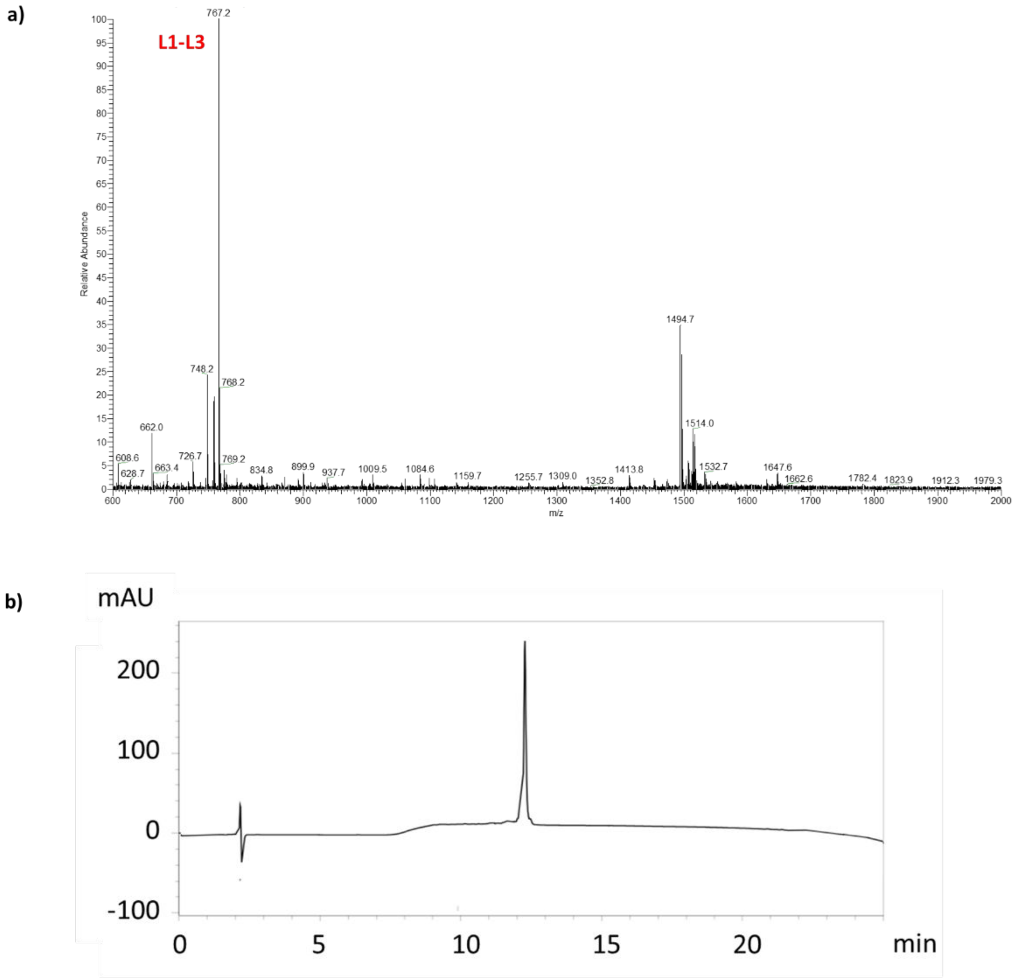

Figure S3: Physicochemical characterization of L1-L3 peptide: a) ESI mass spectrum and b) RP-HPLC chromatography

a)

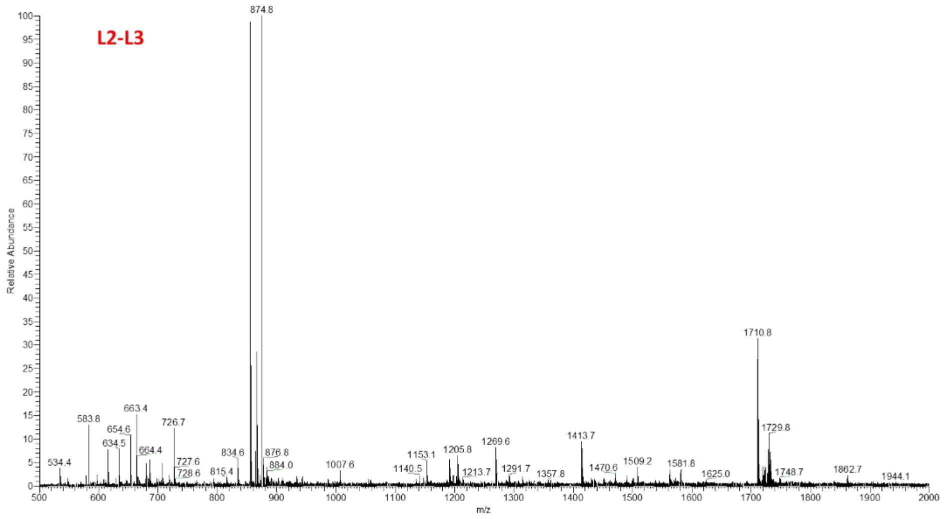

b)

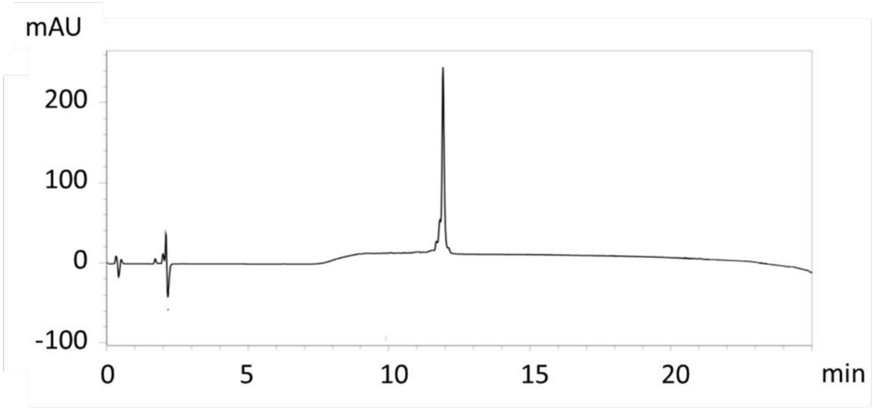

Figure S4: Physicochemical characterization of L2-L3 peptide: a) ESI mass spectrum and b) RP-HPLC chromatography

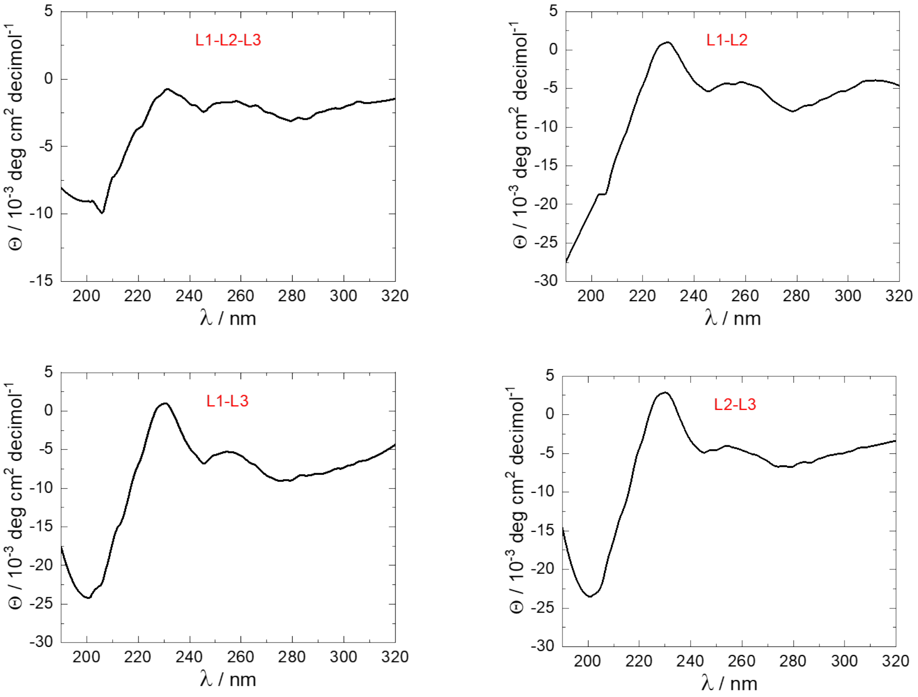

Figure S5: CD spectra of L1-L2-L3, L1-L2, L1-L3 and L2-L3 at a concentration of 2 μM.

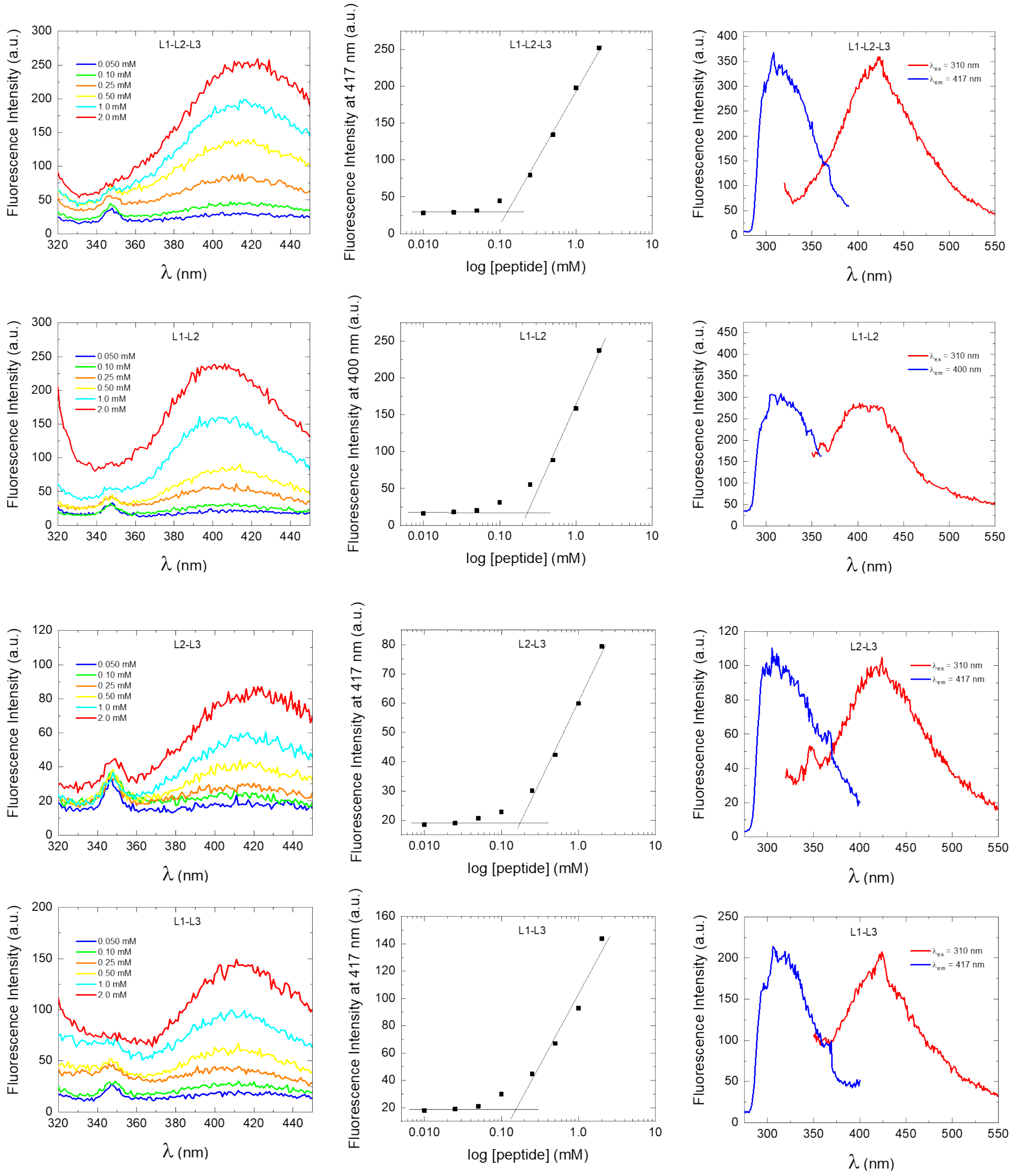

Figure S6: Fluorescence studies of peptides in aqueous solution. Left: Fluorescence emission spectra of peptides as a function of concentration. Center: Fluorescence intensity in the maximum at 417 nm as a function of the peptide concentration. Right: Emission and excitation spectra of peptides at a concentration of 3 mM.

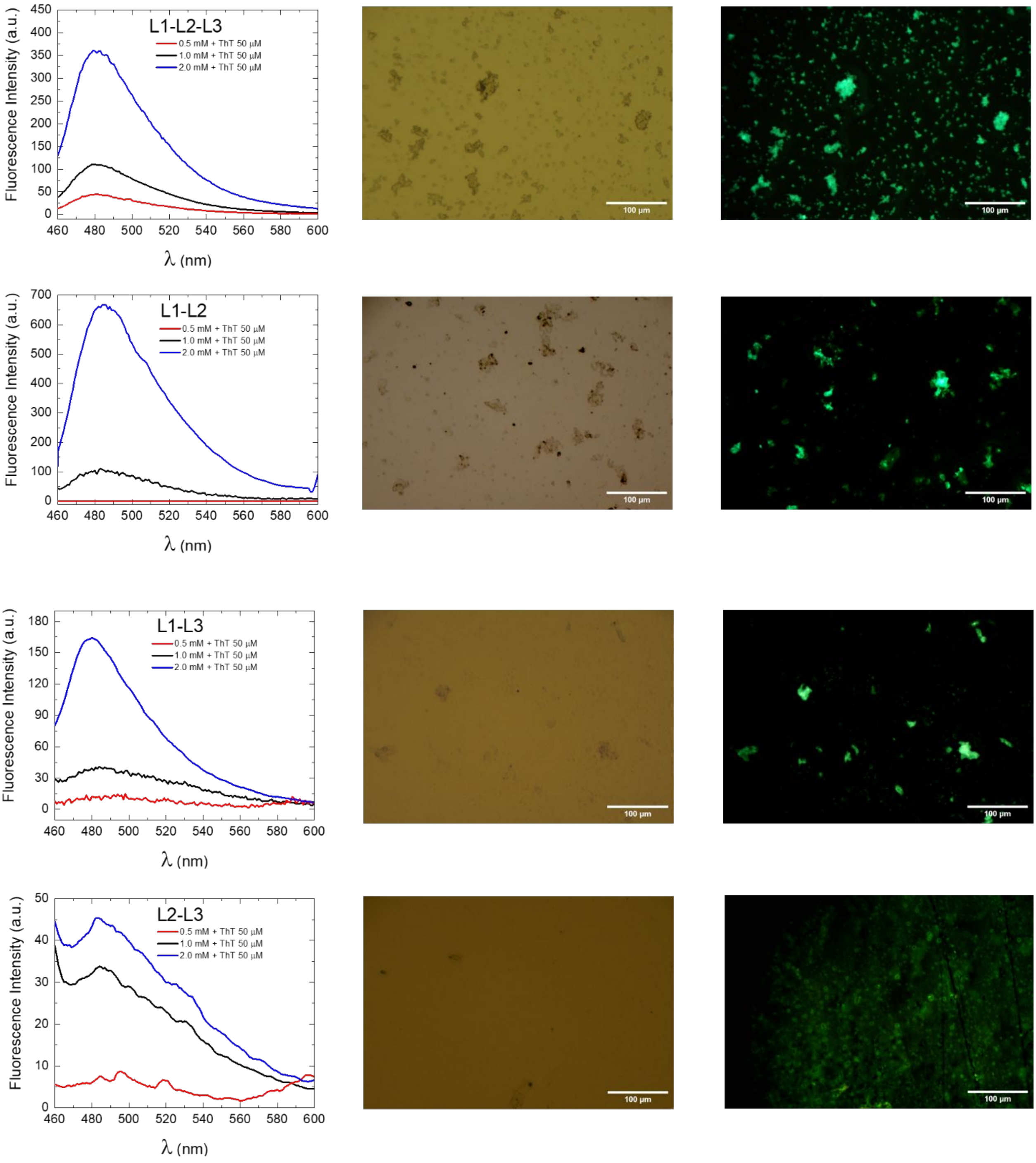

Figure S7: **ThT assay.** Left: Fluorescence emission spectra of L1-L2-L3, L1-L2, L1-L3 and L2-L3 in the presence of ThT at several peptide concentrations in the range 5 μM– 2 mM. Samples were excited at 450 nm and the spectra recorded between 460 and 600 nm. Fluorescence microscopy of peptide aggregates in the presence of ThT: in the center bright field images; on the right fluorescence images excited in the spectral regions of GFP filter (λ_{exc}=488 nm, λ_{em}=507 nm).

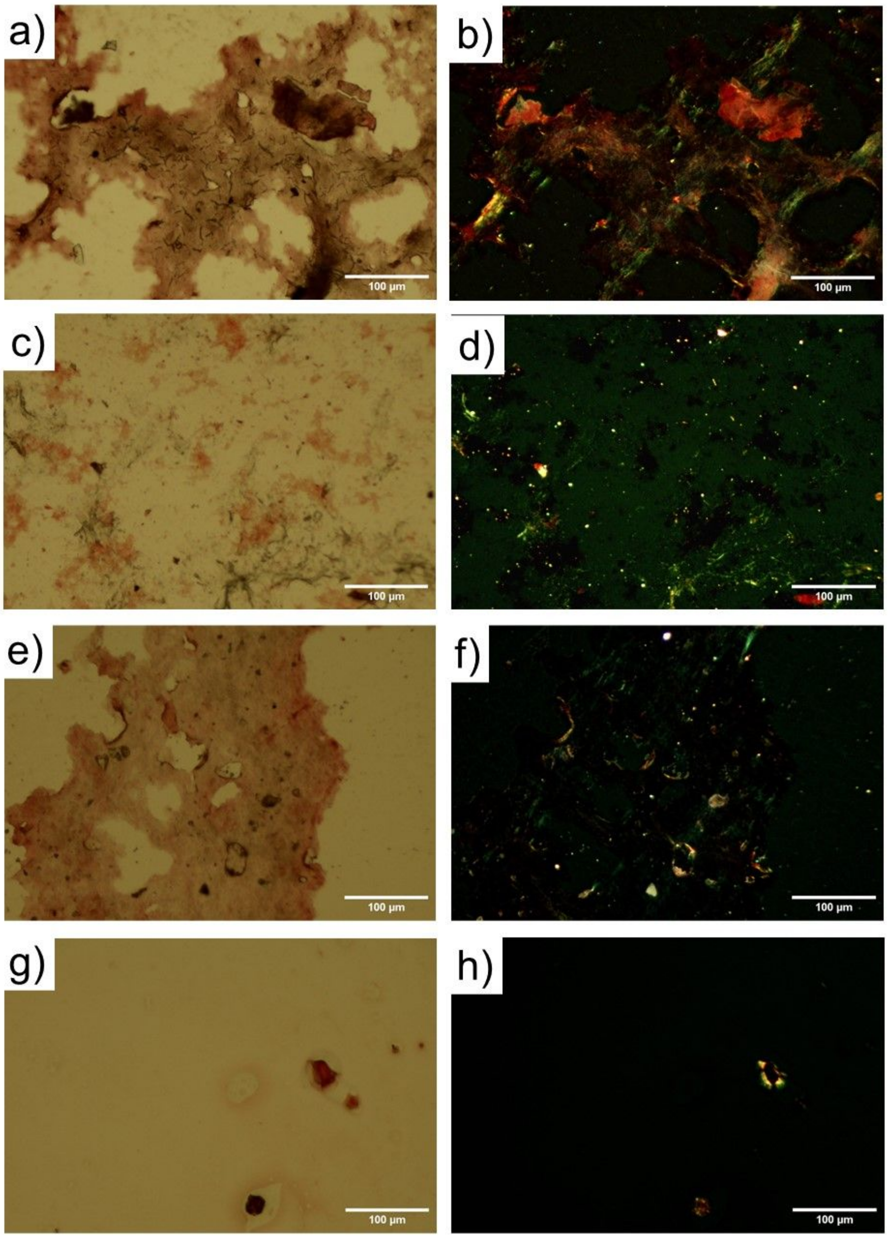

Figure S8: Polarized optical microscopy: images of (a, b) L1-L2-L3, (c, d) L1-L2, (e, f) L1-L3 and (g, h) L2-L3 peptides dried onto a glass slide, stained with Congo red solution and observed with an optical microscope under bright field illumination and between crossed polars, respectively (scale bar 100 µm).

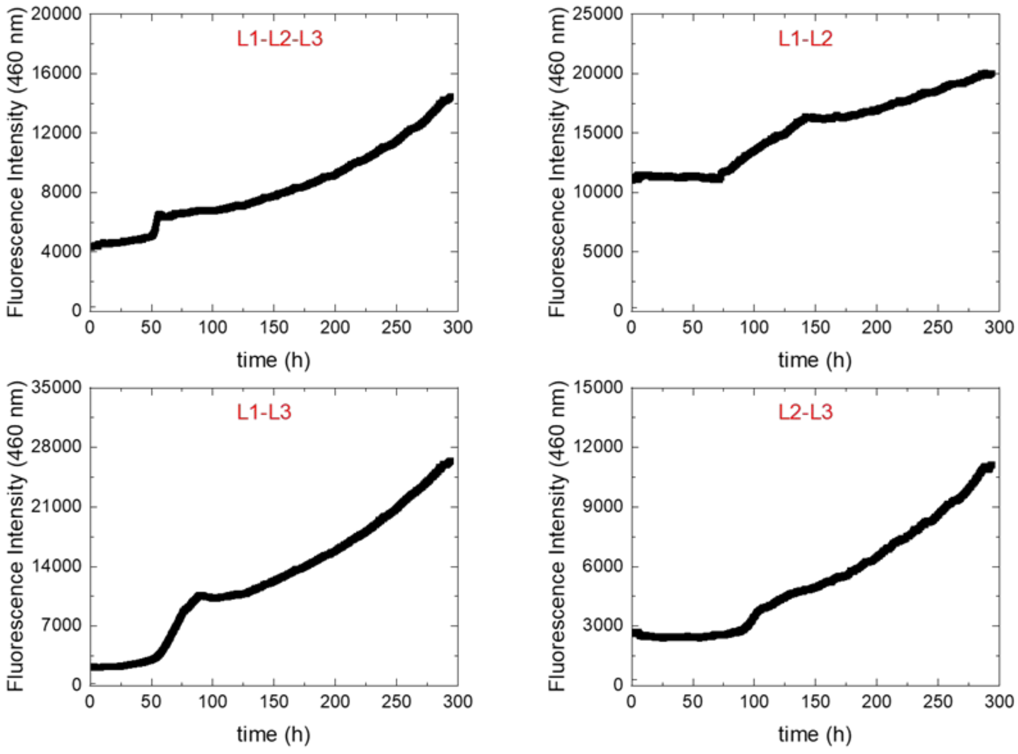

Figure S9: **Aggregation kinetics studies.** Emission intensity at 460 nm (λ_{ex} = 350 nm) of L1-L2-L3, L1-L2, L1-L3 and L2-L3 peptide solutions at 2 mM recorded over 300 h.

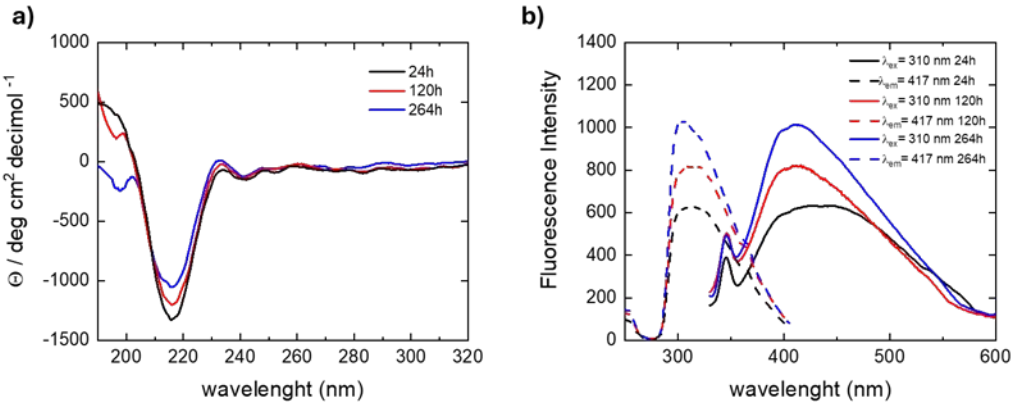

Figure S10: L2-L3 time-dependent CD and fluorescence spectra: a) CD spectra of L2-L3 peptide solutions at 2 mM at 24, 120 an 264h; b) Fluorescence emission and excitation spectra of L2-L3 peptide solutions at 2 mM at the same time points.

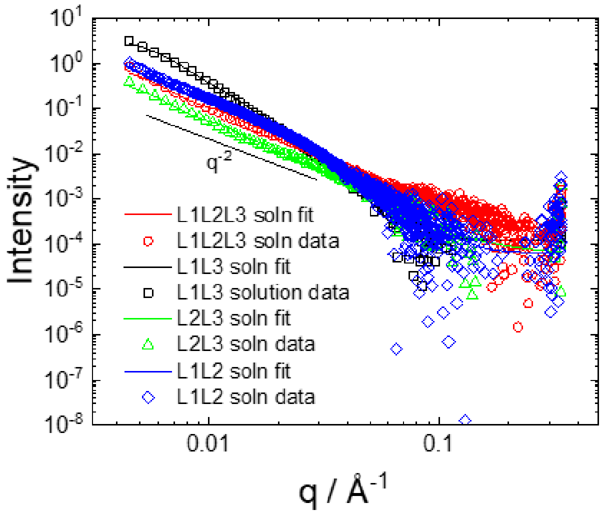

Figure S11: SAXS experiments for solutions. SAXS data and model form factor fits (parameters in Table 3) for solutions in 10 mM PBS. The open symbols are measured data (every 5th data point plotted for clarity) and the lines are the model fits.

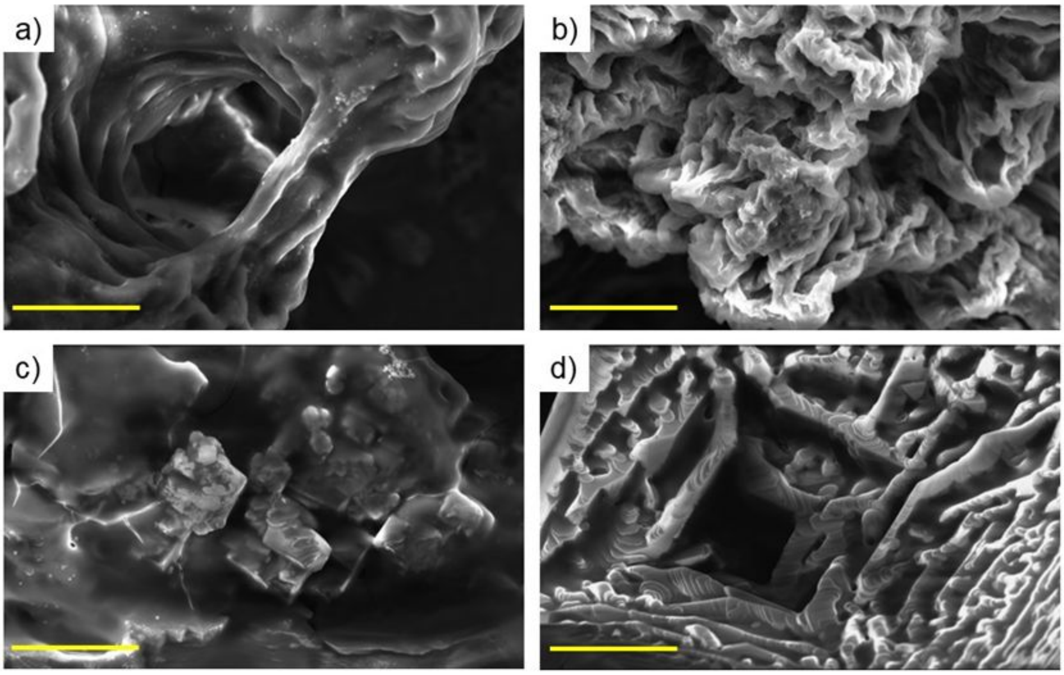

Figure S12: SEM images for solutions. Selected SEM micrographs of peptide solutions at 2 mM concentration: a) L1-L2-L3, b) L1-L2, c) L1-L3 and d) L2-L3. Magnification and scale bar are 6300x and 20 µm, respectively.

a)

L3-L2-L3

Ac-STGGYGGDTSYSSYGQSNGPSTGGYG-NH_2

b)

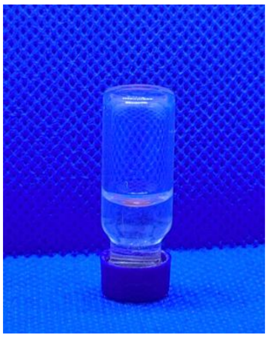

Figure S13: a) L3-L2-L3 sequence; b) inverted test-tube for L3-L2-L3 at 5 wt% concentration.

a)

L1*-L2

Ac-SYGGYSGDTSYSSYGQS-NH_2

b)

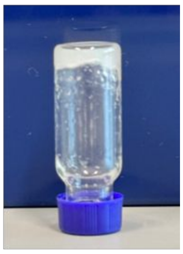

2 wt %

c)

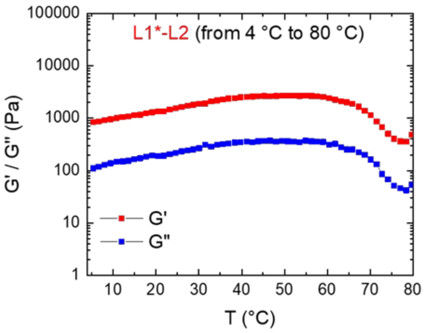

d)

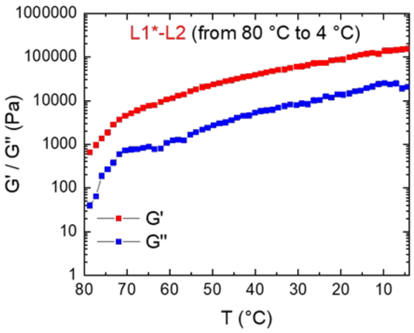

Figure S14: Formulation and characterization of L1*-L2 hydrogel. a) L1*-L2 sequence; b) inverted test tube for L1*-L2 hydrogel at 2 wt% concentration; c) Moduli G' and G'' reported as a function of the temperature increasing from 4 to 80 °C; d) Moduli G' and G'' reported as a function of the temperature decreasing from 80 to 4 °C**.**

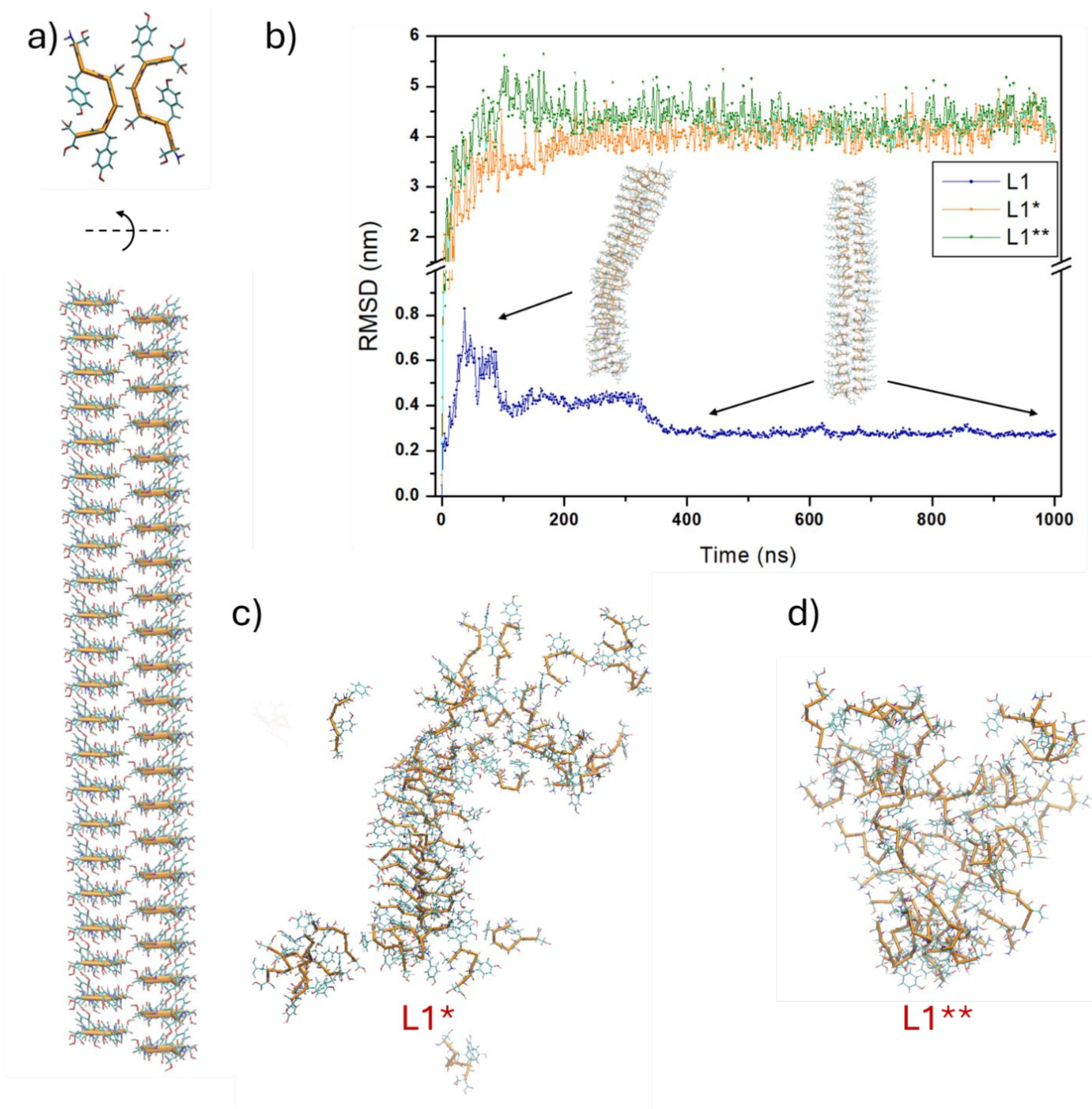

Figure S15: MD simulations of L1 assemblies. a) The stability of the L1 peptide (SYSGYS), L1* (SYGGYS) and L1** (STGGYS) in the assembled structure was evaluated using MD simulations (1 microsecond each) in explicit waters. A steric zipper of a pair of β-sheets of 22 strands was constructed from the crystal structure (PDB code 6BWZ). b) Root means square fluctuations (RMSD) calculated on the Cα atoms of the assembly along the simulations. c) representative structure of L1* assembly during the simulations. d) representative structure of L1** assembly during the simulations.

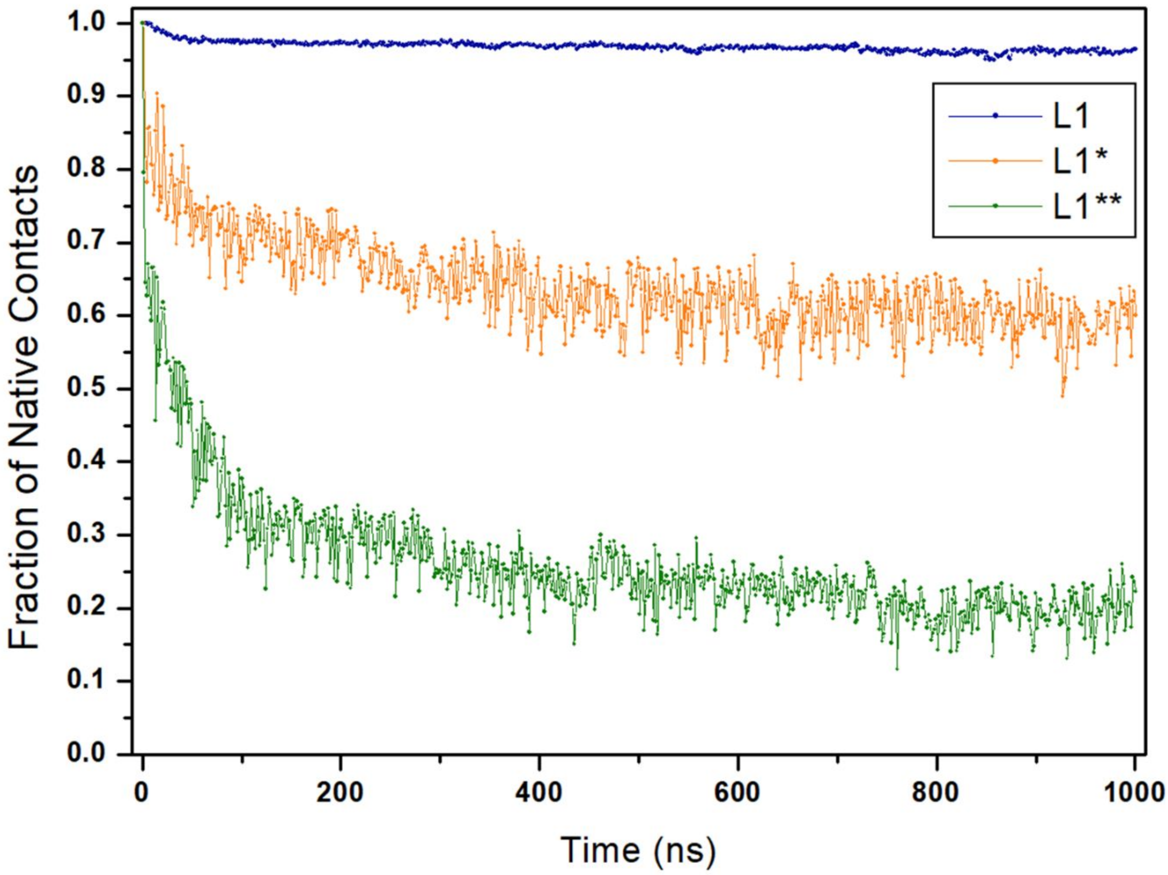

Figure S16: Fraction of native contacts of LARKS assemblies during MD simulations. We evaluated the fraction of preserved native contacts along the simulations. L1 is able to maintain 96.7% of the contacts during the simulations. L1* retains some clusters of the initial structure, but fragmented into smaller units, yielding to a fraction of native contacts of 53% at the end of 1μs simulation. In the case of L1** the fraction of preserved contacts drops to 29%, generating assemblies that resemble condensate phases.
